# Production of zirconium-88 via proton irradiation of metallic yttrium and preparation of target for neutron transmission measurements at DICER

**DOI:** 10.1038/s41598-023-27993-7

**Published:** 2023-01-31

**Authors:** Artem V. Matyskin, Athanasios Stamatopoulos, Ellen M. O’Brien, Brad J. DiGiovine, Veronika Mocko, Michael E. Fassbender, C. Etienne Vermeulen, Paul E. Koehler

**Affiliations:** 1 Chemistry Division, Los Alamos National Laboratory, P.O. Box 1663, Los Alamos, NM 87545 USA; 2 Present Address: Radiation Science and Engineering Center, Pennsylvania State University, 135 Breazeale Nuclear Reactor, University Park, PA 16802 USA; 3 Physics Division, Los Alamos National Laboratory, P.O. Box 1663, Los Alamos, NM 87545 USA; 4 Present Address: Q Division, Los Alamos National Laboratory, P.O. Box 1663, Los Alamos, NM 87545 USA

**Keywords:** Inorganic chemistry, Nuclear chemistry, Chemical engineering, Experimental nuclear physics, Experimental particle physics

## Abstract

A process for the production of tens to hundreds of GBq amounts of zirconium-88 (^88^Zr) using proton beams on yttrium was developed. For this purpose, yttrium metal targets (≈20 g) were irradiated in a ~16 to 34 MeV proton beam at a beam current of 100–200 µA at the Los Alamos Isotope Production Facility (IPF). The ^88^Zr radionuclide was produced and separated from the yttrium targets using hydroxamate resin with an elution yield of 94(5)% (1σ). Liquid DCl solution in D_2_O was selected as a suitable ^88^Zr sample matrix due to the high neutron transmission of deuterium compared to hydrogen and an even distribution of ^88^Zr in the sample matrix. The separated ^88^Zr was dissolved in DCl and 8 µL of the obtained solution was transferred to a tungsten sample can with a 1.2 mm diameter hole using a syringe and automated filling station inside a hot cell. Neutron transmission of the obtained ^88^Zr sample was measured at the Device for Indirect Capture Experiments on Radionuclides (DICER).

## Introduction

Zirconium (Zr) is a group IV transition metal, which has 5 stable and 31 known radioactive isotopes. A few radioactive isotopes of Zr are important to various areas of science and technology. Zirconium-89 (^89^Zr) is one of the most promising radionuclides for immuno-positron emission tomography (immuno-PET) because of its unique physical and chemical properties^1,2^. Its relatively long half-life (78.4 h) matches the biological half-life of antibodies and antibody fragments and it decays to stable yttrium-89 (^89^Y) via electron capture (77%) and positron emission (23%) emitting mostly 511 keV gamma rays from annihilation, 909 keV gamma rays and a few X-rays^3^. Besides this, significant amounts of ^89^Zr can be relatively easily produced with a low energy proton beam (E_p_ < 13.1 MeV) onto a monoisotopic ^89^Y target and the produced ^89^Zr can be efficiently separated from the target, chelated and attached to the antibody^4^.

Another interesting isotope of zirconium is ^88^Zr, which has a half-life of 83 days and decays to ytrrium-88 (^88^Y) via electron capture, emitting 393 keV gamma rays and a few x-rays. Yttrium-88 (t_1/2_ = 106.6 days) decays to stable strontium-88 (^88^Sr) mainly via electron capture, emitting gamma rays of 898 keV and 1836 keV and a few x-rays^3^. Thus, ^88^Zr can be used to produce high-purity, carrier-free ^88^Y in a radionuclide generator system. Both ^88^Zr and ^88^Y are useful tracers in radiopharmaceutical research as longer-lived surrogates of promising immuno-PET ^89^Zr^5^, and in radioimmunotherapy and radioembolization therapy with ^90^Y^6^, respectively.

Natural zirconium was widely used in nuclear devices during nuclear weapon testing as a loaded detector material or radiochemical diagnostic i.e., it was used to derive a neutron fluence from the initial amount of loaded Zr and measured activities of the Zr isotopes formed in the neutron environment^7^. The neutron fluence derived from the experimental and historical data can be compared with the neutron fluence computed using various codes, which use neutron-induced cross sections. Zirconium-88 is one of the most important Zr isotopes formed in these high-energy neutron induced reactions^8^ and precise measurements of its (n,γ) cross section can be used to improve the codes and thus get a better understanding of the device performance. Moreover, recently Shusterman and co-workers found that ^88^Zr has an unexpectedly high thermal neutron capture cross section of (8.61 ± 0.69)·10^5^ barns^9^. Presumably, the large thermal neutron cross section of ^88^Zr is caused by one or more low energy resonances. A detailed study of the neutron capture cross section of ^88^Zr over a large energy range is needed to determine the properties of its extremely high thermal neutron capture cross section and to obtain the first point-wise experimental data at neutron energies up to the keV range to inform the accuracy of neutron fluence codes. Such a study would have an impact both on fundamental and applied levels.

The Device for Indirect Capture Experiments on Radionuclides (DICER) is a novel neutron transmission instrument which was conceived, designed and developed at the Los Alamos Neutron Science Center (LANSCE). It can be used to measure and tightly constrain (n,γ) cross sections over a wide energy range by performing neutron transmission measurements through the sample as a surrogate to direct neutron capture studies. DICER is especially useful for measurements of neutron-capture cross sections of small amounts of highly radioactive radionuclides, like ^88^Zr. This is because typical sample-to-detector distances in transmission set-ups like DICER are of the order of tens of meters as opposed to a few dm in direct neutron-capture experiments. Therefore, transmission experiments suffer appreciably less from the intrinsic ^88^Zr decay background, while the close sample-detector proximity in direct (n,γ) measurements on radionuclides renders them challenging. Several other indirect techniques for (n,γ) cross section determination have been already developed, such as the surrogate^10^, γ-ray strength function^11,12^, Oslo^13–16^ and β-Oslo^17^ methods. Even though all these methods were found to be useful, they are very dependent on theory and therefore, values of cross sections obtained have very high uncertainties. The DICER technique measures the same neutron resonances that determine the neutron-capture cross section experimentally. Therefore, the DICER method is less dependent on theory and hence should be more accurate than other indirect techniques.

The goal of the present work was to produce tens to hundreds of GBq amounts of ^88^Zr by proton irradiation at the Los Alamos Isotope Production Facility (IPF)^18^, separate the produced ^88^Zr from the target material and impurities and prepare a ^88^Zr target suitable for neutron transmission measurements at DICER.

## Methods

All experimental work with the irradiated Y target was performed in the hot cells. All the methods described including target dissolution, separation of µg amounts of Zr from gram amounts of Y and preparation of Zr target for neutron transmission measurements were first developed and tested with non-radioactive samples.

### Chemicals and analytical measurements

All solutions were prepared using an analytical balance. All chemicals used in this work are listed in Table 1.

**Table 1:** Chemicals used.

Chemical	Source	Comment
Yttrium metal targets	Alfa Aesar	99.9% purity
Hydroxamate resin	TrisKem	50–100 μm
Oxalic acid	MilliporeSigma	99.999% trace metals basis
HCl	MilliporeSigma	99.999% trace metals basis
HCl	Fisher Scientific	Optima grade
HNO_3_	Fisher Scientific	Optima grade
H_2_O	Merck Millipore®	18.2 MΩ·cm at 25 °C
H_2_O	Merck Millipore®	Teflon distilled
D_2_O	Acros Organics	Minimum 99.95% deuterium
DCl	Acros Organics	Minimum 99.95% deuterium

Quantitative radiometric measurements were performed using a High Purity Germanium Detector (Ortec GEM30P4-76, coaxial, *p*-type HPGe detector, 61.9 mm diameter, 43.4 mm length and 0.7 mm Ge/Li dead layer) with a relative efficiency of 31% and resolution (FWHM) of 1.75 keV at 1332.49 keV. The HPGe detector was cooled with liquid nitrogen (Ortec Möbius Recycler system) and coupled to a digital spectrum analyzer (Ortec DSPEC502). The detector was calibrated in the energy interval of 60–1836 keV for the same geometry (5 mL aqueous solution in 20 mL, plastic liquid scintillation vial) using a mixed radionuclide reference solution (NIST traceable from Eckert and Ziegler, USA). Daily calibration checks were performed with a ^152^Eu source (Eckert and Ziegler, USA). Counting dead time was always kept below 10%.

Qualitative radiometric measurements of load solutions, eluates, residues, glass- and plasticware etc. were performed directly from the hot cell (cross-corridor measurements) using another HPGe detector (Ortec GEM10P4-70-PL, coaxial, *p-*type HPGe detector, 56.4 mm diameter, 29.2 mm length and 0.7 mm Ge/Li dead layer) which has relative efficiency of 14% and resolution (FWHM) of 1.57 keV at 1332.49 keV. The HPGe detector was cooled with liquid nitrogen (Ortec Möbius Recycler system) and coupled to a digital spectrum analyzer (Ortec DSPEC502).

All gamma spectra were evaluated using the Gamma Vision 7.01.03 software. All half-life, gamma emission energies and photon emission probabilities were taken from the Decay Data Evaluation Project^3^.

Concentrations of stable elements in the first target were measured by Inductively Coupled Plasma Optical Emission Spectroscopy (ICP-OES) using a Perkin-Elmer Optima 8000. The instrument was calibrated using various NIST traceable calibration standards, which were prepared in the same matrix as the measured ^88^Zr sample. Quality checks were performed a few times between measurements using sample with 0.2 mg·L^−1^ of Zr and Y, prepared using different standards from those using for calibration. The following elements were measured, and wavelengths used: Zr (339.197 nm), Ca (396.847 nm), Fe (259.939 nm), Ti (334.94 nm), Ta (240.063 nm), Y (371.029 nm), Al (396.153 nm). The data were evaluated using WinLab32 ICP software.

### Proton irradiation of yttrium metal target

Two yttrium (Y) metal targets were encapsulated in a standard aluminum bolt-together target capsule (~1 mm window) and third target was encapsulated in Inconel capsule (~0.5 mm window). All targets were irradiated in the low energy target position at the Isotope Production Facility (IPF)^18^. A total of three irradiations were conducted, the first in December 2020, the second in June 2021 and the third in August 2022. Target and irradiation parameters are listed in Table 2, including the total integrated charge received.

**Table 2:** Yttrium metal targets and irradiation parameters.

	Yttrium metal target 1	Yttrium metal target 2	Yttrium metal target 3
Target mass (g)	21.009	19.5926	26.94
Diameter and thickness (mm)	45.99 × 2.9	43.1 × 2.99	50.8 × 3
Irradiation time (h)	8.93	34.43	126.48
Incident proton energy (MeV)	34.3	34.3	32.7
Average beam current (µA)	96.1	100	200
Total integrated beam (µAh)	858.53	3002.1	18532

The total integrated beam current shown in Table 2 is a function of the total received beam over the duration of the irradiation. For the second target irradiated, there was some period of time over which the proton beam was off, leading to a longer irradiation time in order to reach the required integrated current of ~3000 µAh.

The desired proton energy window was obtained by using two aluminum degraders upstream of the Y target material in order to reduce the average proton beam energy to the desired ~34 MeV incident energy on the Y target.

### Dissolution of proton irradiated Y metal target

After the irradiations all targets were transported to the LANL radiochemistry facility and disassembled or opened inside the hot cell. The Y metal disk in the second target was partly cracked. All targets were dissolved in ≈150 mL of 6 mol·L^−1^ HCl by slow addition of ≈10 mL portions. Formation of an insoluble black residue was observed in all cases and obtained suspensions were first allowed to settle, decanted and then filtered using a 0.45 µm membrane cellulose nitrate filter. The filtrate and filter with the black residue were measured via HPGe detector and it was found that the ^88^Zr and ^88^Y count rates from the filter are significantly lower (≈1%) compared to count rates from the filtrate. Similar observations and conclusions were made by Holland and co-workers^19^. The filter with the black residue was discarded and a small aliquot was taken from the filtrate and measured via HPGe.

### Separation of ^88^Zr from irradiated yttrium metal target using hydroxamate column

Two grams of hydroxamate resin were loaded into a custom, in-house made polypropylene column: 1.5 cm diameter, 11.5 cm working bed height, 30 µm polyethylene filter with 100 mL reservoir on the top. This column was used for gravity-flow chromatography and the loaded column was washed first with water and then with 2 mol·L^−1^ HCl. The filtrate with dissolved Y metal target was diluted with water from 6 to ≈2 mol·L^−1^ HCl and the obtained solution was passed through the column. The eluate was collected in 50 mL portions and every portion was measured via a HPGe detector to control the ^88^Zr breakthrough. After the ^88^Zr load, the column was washed with ≈50 mL of 2 mol·L^−1^ HCl to remove any possible Y. Then, the ^88^Zr was eluted via three 10 mL portions of 1 mol·L^−1^ C_2_H_2_O_4_.

Almost all ^88^Zr was eluted via the first 10 mL of 1 mol·L^−1^ C_2_H_2_O_4_ and 15 mL of 15.5 mol·L^−1^ HNO_3_ was added to this eluate to decompose oxalic acid according to the reaction^20,21^:

$${\text{3H}}_{{2}} {\text{C}}_{{2}} {\text{O}}_{{4}} + {\text{ 2HNO}}_{{3}} \rightleftharpoons {\text{6CO}}_{{2}} + {\text{ 2NO }} + {\text{ 4H}}_{{2}} {\text{O}}$$

This procedure was repeated 2 more times to ensure complete decomposition of oxalic acid. After that, ^88^Zr in 15.5 mol·L^−1^ HNO_3_ was evaporated down to ≈2 mL and ≈10 mL of 6 mol·L^−1^ HCl added.

### Preparation of the ^88^Zr target for neutron transmission measurements

The matrix of the ^88^Zr sample was changed from HCl to DCl by evaporation of the sample down to ≈2 mL and addition of DCl in D_2_O. The obtained sample was evaporated again down to ≈1 mL and another 10 mL of DCl in D_2_O was added and the procedure repeated one more time. The final ^88^Zr solution from target 2 was evaporated to obtain 0.1041 g of ≈2 mol·L^−1^ DCl in D_2_O. Zirconium-88 solution from target 3 was also dissolved in ≈2 mol·L^−1^ DCl in D_2_O and formation of hardly visible precipitate was observed.

### Activation estimations

Estimations of the anticipated activities produced were calculated to determine the optimum energy window, target thickness, and required integrated proton current for production of the required quantities of ^88^Zr. These estimations were done computationally, using the known IPF target station and Y target geometry, the traditional stopping power formulas, and available model predicted cross section data. The formula for the determination of integrated reaction rate of a target of some thickness *x*, from which the total activity produced can be extracted over a known integral energy range and irradiation time, is provided below.

$$R={I}_{beam}{\int }_{{E}_{min}}^{{E}_{max}}\frac{\mathrm{N\sigma }(\mathrm{E})}{\rho }\left(\rho /\frac{dE}{dx}\right)dE$$

where $\rho /\frac{dE}{dx}$ is the inverse of the mass stopping power, ${I}_{beam}$ is the beam intensity, *N* is the material number density, and $\upsigma (\mathrm{E})$ is the microscopic cross section describing the probability of generating a specific isotope in a given target material at a specific proton energy. From this reaction rate, a total activity can be determined.

### Uncertainty assessment

Stochastic (Type A) uncertainties of all gamma-spectrometric measurements (1σ) were usually between 3 and 4%. These uncertainties were combined with the uncertainties of volumetric measurements (inside the hot cell) and propagated to the uncertainties of EOB activities. HPGe measurements of the filtrate from dissolved Y metal targets and filter with the black residue showed that activities of radionuclides in the residue is approximately 1% compared to the activities of these radionuclides in the filtrate. These uncertainties were classified as systematic (Type B), and EOB activities of all radionuclides were increased by 1% to account for the activity losses in the black residue. Uncertainties in the ^88^Zr elution (1σ) from the hydroxamate column were calculated from duplicates (two independent experiments with target 1 and 2). Uncertainties in ICP-OES measurements were calculated from 2 different aliquots taken from the same sample.

## Results and discussion

### Targetry for ^88^Zr production in a proton beam

There are many nuclear pathways to produce ^88^Zr and comprehensive reviews of common production routes are available^22–24^. These methods include irradiation of natural Mo metal, natural monoisotopic Nb metal, natural Zr metal or natural Y (either in metallic or oxide forms). Some other, more exotic methods of ^88^Zr production include ^88^Zr recovery as a by-product from irradiated Nb capsules used as target cladding^25,26^ and possible ^88^Zr harvesting at the Facility for Rare Isotope Beams (FRIB)^27^. Given the proton energies and the large ^89^Y(p,2n)^88^Zr production cross section of nearly 1 barn, the method which can be used to produce high amounts of carrier-free ^88^Zr at IPF is irradiation of natural, monoisotopic yttrium in metal or oxide form.

**Figure 1:**
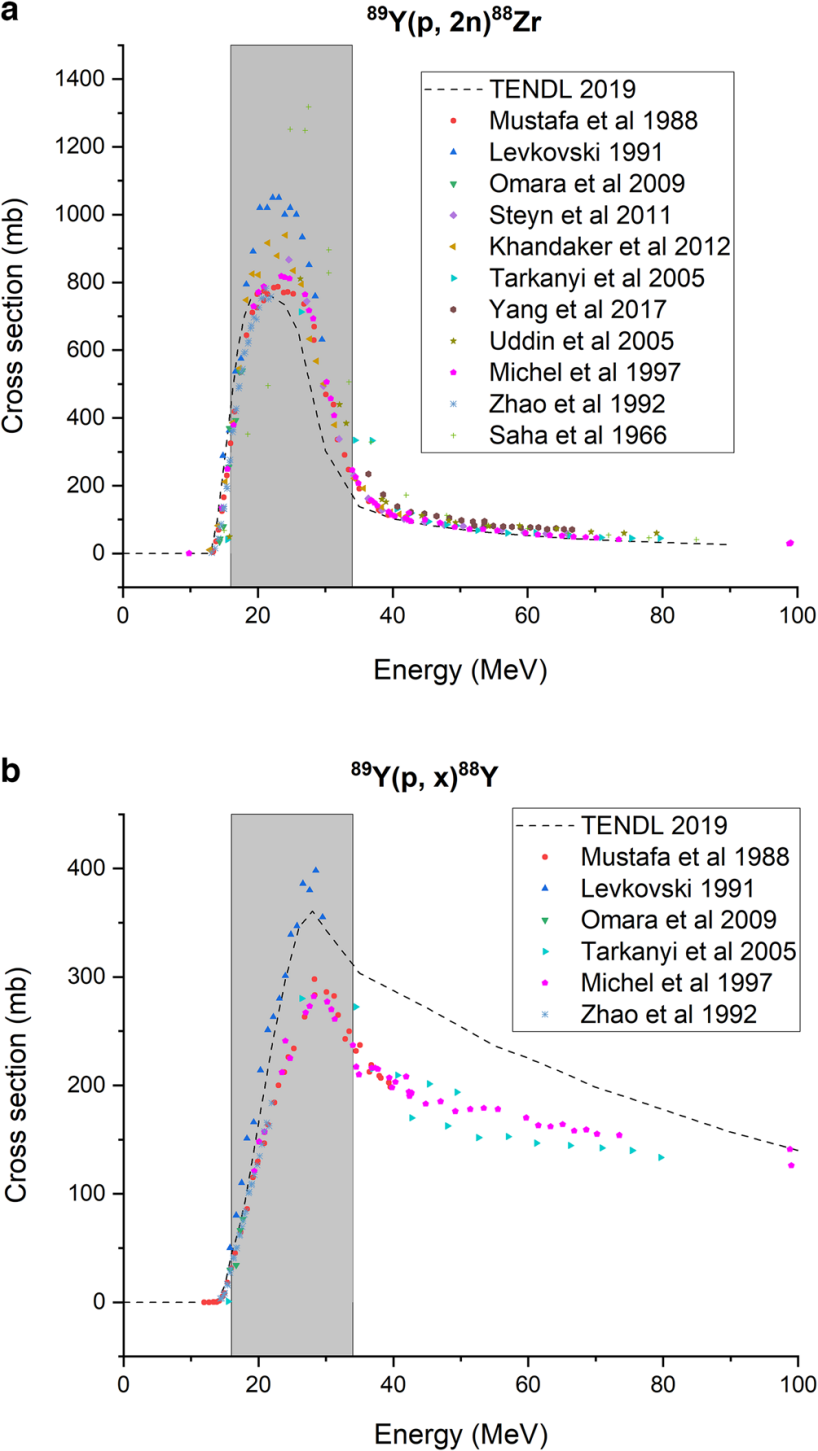
(**a**) Experimental literature data^23,28–37^ (points) and model predicted TENDL 2019 data^38^ (dotted line) for the ^89^Y(p,2n)^88^Zr cross section and the predicted Y target energy window (gray). (**b**) Experimental literature data^23,29–32,37^ (points) and model predicted TENDL 2019 data^38^ (dotted line) for the ^89^Y(p,x)^88^Y cross section and the predicted Y target energy window (gray).

A stacked target configuration was utilized in the IPF target station that allowed for the Y target material to capture the peak of the ^88^Zr production cross section. Literature experimental data and model predictions for the ^89^Y(p,2n)^88^Zr and ^89^Y(p,x)^88^Y cross sections and the Y target energy windows predicted using the IPF upstream target stack configuration and stopping power calculation are shown in Fig. 1a,b, respectively.

As shown in Fig. 1a, there is good agreement between experimental^23,28–37^ and TENDL 2019 data^38^ for the ^89^Y(p,2n)^88^Zr cross section, which is typical for the (p,2n) reactions, as well as for the (p,n) reactions like ^89^Y(p,n)^89^Zr. Only data from Levkovski^29^ are approximately 100–200 mb higher in the 15–30 MeV energy range. Levkovski^29^ used highly enriched targets to measure proton-induced cross-section data and obtain excitation functions for approximately 300 reactions. He used the ^nat^Mo(p,x)^96^Tc reaction to monitor the beam intensity and the cross-section value of 250 mb at 30 MeV for this reaction. Later it was found that almost all the cross-section data obtained by Levkovski^29^ are systematically higher and it was explained by the use of too high ^nat^Mo(p,x)^96^Tc monitor cross-section data^39^. Therefore, Takacs et al.^39^ and Qaim et al.^40^ proposed to use 0.8 and 0.82 correction factors for the Levkovski data^29^, respectively. Contrary, as shown in Fig. 1b, almost all experimental data for the ^89^Y(p,x)^88^Y cross section are approximately 100 mb lower compared to the TENDL 2019 data^38^ in the 25–35 MeV energy range and only data from Levkovski^29^ fit TENDL 2019 data^38^ quite well. As discussed above, Levkovski cross-section data^29^ are subject to systematic uncertainties and should be reduced by approximately 20%.

Either metallic yttrium or yttrium oxide could be chosen as a target material. For the same target dimensions and energy window, metallic Y is preferable because there are more Y atoms per target unit area and, as a result, higher integral thick target yields of ^88^Zr. However, metallic Y is chemically aggressive and fine particles are pyrophoric, therefore it is difficult to manufacture Y metal targets via powder compression. Bulk Y metal is stable in air due to passivation of its surface (formation of Y_2_O_3_). Finally, the purity of the initial target material is of major importance, because even small amounts of stable impurities in the target could result in high activities of co-produced impurity radionuclides if the cross section is large. Yttrium oxide with a very high purity (99.999%) is widely available, while the availability of high purity Y metal is limited because it is chemically aggressive and difficult to synthesize. For example, one of the methods used to obtain metallic Y is the reduction of YF_3_ in a tantalum crucible at 1550 °C by Ca metal in argon atmosphere and the reduced metal usually contains some Ca (≈0.02 wt%), Ta (≈0.3 wt%), Fe (≈0.05 wt%), rare earth (≈0.1–0.2 wt%), as well as some non-metallic impurities. Further purification of Y metal includes its subsequent melting in vacuum and results in Y separation from Ca, Ta and Fe (< 0.01–0.001 wt%). The separation of metallic Y from some rare earth elements and some non-metallic impurities (H, C, O, N) is very difficult^41^. Both Y metal and Y_2_O_3_ can be dissolved in relatively diluted HCl (≈2 mol·L^−1^).

As discussed before, ^89^Zr is a promising immuno-PET radionuclide and therefore its production has been studied by many authors. Similar to ^88^Zr, high amounts of ^89^Zr can be produced via irradiation of either Y metal or Y_2_O_3_ in a proton beam but at lower proton energies. Usually, only small targets (1 g or less) have been used to produce GBq (mCi) amounts of ^88^Zr or ^89^Zr and some authors selected yttrium metal^9,19,42–49^ (typically sputtered on Nb or Cu) or pressed yttrium oxide^50–52^ as a target material. Liquid yttrium nitrate targets have also been studied^42^ for the production of ^89^Zr.

In this work, metallic yttrium was chosen as the target material and thick yttrium metal sputtering targets (≈20 g) were placed inside a bolted aluminum target-holder or enclosed in an Inconel capsule and irradiated in a high intensity proton beam to explore the feasibility of ^88^Zr production of tens to hundreds of GBq amounts.

### Dissolution of proton irradiated Y metal targets and measurement of target yields

After irradiation, the targets were delivered to the LANL hot cell facility and short-lived radioactive impurities were allowed to decay. All targets (≈20 g each) were dissolved in ≈150 mL of 6 mol·L^−1^ HCl by slow addition of ≈10 mL aliquots and dissolution took ≈30 min. Some authors used less concentrated HCl, for example Mejs et al.^44^ dissolved a small irradiated Y metal sputtered target on Cu backing in 1 mol·L^−1^ HCl at room temperature and added H_2_O_2_ to ensure complete oxidation of Zr. Other authors also used relatively dilute 1 mol·L^−1^ HCl to dissolve small Y metal targets^43,47–49^ and slow dissolution usually followed by evaporation to dryness and redissolution in the desired media. Queern et al.^45^ used more concentrated 2 mol·L^−1^ HCl and 80 °C to speed up the dissolution process. Another commonly used procedure was developed by Holland and co-workers^19^ and includes dissolution of the Y metal target in 6 mol·L^−1^ HCl at room temperature and addition of H_2_O_2_^42,46^. Finally, concentrated HCl (10 mol·L^−1^ or more) is also sometimes used to dissolve Y metal or Y_2_O_3_ targets^50,51^, in this case ^88^Zr or ^89^Zr is separated from stable Y using an anion exchange resin. In this work, preliminary experiments with the stable Y metal showed that the optimal HCl concentration for massive (≈20 g) Y target dissolution is 6 mol·L^−1^ or higher. Lower concentrations of HCl (2–6 mol·L^−1^) can also be used but in those cases the dissolution process takes more time, while dissolution of a massive Y metal target in 1 mol·L^−1^ HCl is difficult. These results are in excellent agreement with the results obtained by Holland and co-workers^19^. Therefore, a similar but scaled up dissolution procedure was used in this work with the exception that hydrogen peroxide was not added.

After dissolution of all targets in 6 mol·L^−1^ HCl, we observed formation of a black insoluble residue, which was separated from the liquid phase and measured via an HPGe detector. It was found that the residue contains an insignificant amount of ^88^Zr and ^88^Y (≈1%) compared to the amount of ^88^Zr and ^88^Y in the liquid phase. Unfortunately, the formation of insoluble residues is not usually discussed in the literature, and such observations were reported only by a few authors. For example, Holland and co-workers^19^ reported formation of a black insoluble residue when an irradiated Y metal target (0.33 g) was dissolved in 2 mL of 6 mol·L^−1^ HCl at room temperature. The authors hypothesized that the black residue is an insoluble form of yttrium chloride, while we think that it is yttrium hydride (YH_x_ where x is equal to 1, 2 or 3), which is probably formed during proton irradiation of metallic yttrium. Yttrium hydrate is a black compound which is insoluble in aqueous media. Based on the results of this work, we have concluded that this residue contains almost no Zr and this conclusion is in good agreement with the results of Holland et al.^19^. This black residue was discarded, and a small aliquot was taken from the filtrate and measured via HPGe. Activities of all the radionuclides were measured via HPGe detector after 86 days (target 1), 10.42 days (target 2) and 6.21 days (target) 3 of cooling, recalculated back to the end of bombardment (EOB) and corrected for the dilution factor of the aliquot and compared to yields estimated using TENDL 2019 model predicted cross section data^38^ (Table 3), with the exception of ^87^Y, which is presented at the measurement date and time.

**Table 3:** Measured, scaled and model predicted End of Bombardment (EOB) activities of radionuclides in dissolved Y metal targets.

Nuclide	Gamma emissions (keV)	Half-life (days)	Target 1 aliquot measured activity (kBq)	Target 1 scaled EOB activity (GBq)	Target 1 predicted EOB activity (GBq)	Target 2 aliquot measured activity (kBq)	Target 2 scaled EOB activity (GBq)	Target 2 predicted EOB activity (GBq)	Target 3 aliquot measured activity (kBq)	Target 3 scaled EOB activity (GBq)	Target 3 predicted EOB activity (GBq)
^88^Zr	392.9	83	96 (3)	7.9(3)	8.3	11.8(4)	22(1)	28.9	22.6(7)	181(6)	176.8
^89^Zr	909 1713.1 1744.7 1657.6 1620.8	3.27	–	–	51.9	9.6(3)	155(5)	166.3	28.9(9)	820(26)	753.2
^88^Y	1836.1 898 850.6	106.63	90(4)	2.3(1)	3.5	4.0(2)	5.4(3)	10.7	7.4(3)	50(2)	67.8
^87^Y	388.5 484.8	3.33	–	–	24.6	4.3(1)	8.9(2)*	49.7*	14.0(5)	104(4)*	235.3*
^48^V	983.5 1312.1 944.1	15.97	–	–	–	0.073(2)	0.19(2)	–	0.11(3)	1.1(3)	–

*Listed measured and predicted activities of ^87^Y are at the time of measurement. Uncertainties in brackets are 1σ standard deviations. Total integrated beam for Target 1: 858 µAh, Target 2: 3002.1 µAh, Target 3: 18532 µAh. Dilution factor and time elapsed from EOB until the HPGe measurement for Target 1: 39400 and 86 days; for Target 2: 1750142 and 10.42 days; for Target 3: 7520632 and 6.21 days.

As shown in Table 3, 7.9(3) GBq, 22(1) GBq and 181(6) GBq of ^88^Zr was produced at EOB from the first, second and third target, respectively. EOB activity of ^88^Zr was 95% of the predicted value for the first target, 76% for the second target and 102% for the third target. The EOB activities of ^89^Zr in the second and third target were 95% and 109% of the predicted value, respectively. Zirconium-89 was not detected in the first target assay due to the long cooling time (86 days). Measured EOB activities of both ^88^Zr and ^89^Zr are in good agreement with the predicted values, as expected.

The significant activity of ^88^Y present in the sample from the first target (90 kBq, Table 3) is the result of long cooling times and ^88^Zr decay into ^88^Y. The measured activities of ^88^Y back calculated to EOB in the first, second and third target were corrected for the ^88^Zr decay (Table 3) and are only 66%, 51% and 74% of the predicted values, respectively. This discrepancy can be explained by the fact that TENDL 2019 ^89^Y(p,x)^88^Y cross section data^38^, which were used for estimations, are significantly higher than the experimental data in the energy window used (Fig. 1b).

Another yttrium isotope, ^87^Y, was also measured in the second and third target assays (Table 3). Given the long decay period of the first target prior to processing and activity quantification, this isotope had fully decayed away prior to assay and thus there is no data presented on this isotope for target 1. Similar to ^88^Y, ^87^Y is produced both directly in the proton beam and from the decay of parent radionuclides — ^87^Zr and ^87m^Y. Both parent radionuclides are also produced directly. The activities of ^87^Zr and ^87m^Y in the measured samples were below the detection limit, since they are both short-lived (^87^Zr t_1/2_ = 1.68 h, ^87m^Y t_1/2_ = 13.37 h) relative to the total decay time prior to measurement. Therefore, the measured ^87^Y activities presented in Table  3 were not projected back to EOB and only reflect the activity at time of measurement including both decay of ^87^Y produced directly and ^87^Y feeding via decay of ^87^Zr and ^87m^Y. Predicted activities of ^87^Y were calculated by folding in the quantity of ^87^Y produced at EOB and decay and subsequent ingrowth due to decay of ^87^Zr and ^87m^Y to the date and time of the measurements. As shown in Table 3, measured activity of ^87^Y is 18% and 45% from the predicted activities from target 2 and 3, respectively. As the energy window in the targets was located at the threshold for the ^87^Y and parent reactions, uncertainties in the proton energy loss and straggle as well in the TENDL cross sections and cross section energy thresholds could contribute to the discrepancies.

The presence of vanadium-48 (t_1/2_ = 15.97 days) is most probably the result of the ^48^Ti(p,n)^48^ V reaction. Titanium-48 is the most abundant (73.7%) natural isotope of titanium and was probably present in the Y metal target as an impurity. Similar results were obtained by Meijs and co-workers^44^, who irradiated an Y metal sputtering target in a proton beam (1 h, 100 µA) to produce 4.8 GBq of ^89^Zr. They measured ^56^Co, ^65^Zn and ^48^ V in the dissolved Y metal target. The presence of ^48^V (t_1/2_ = 15.97 days) and ^56^Co (t_1/2_ = 77.24 days) was also explained by the (p,n) reaction on natural Fe and Ti target impurities, respectively, and confirmed by p-induced X-ray emission spectrometry of the target material.

### Separation of ^88^Zr from irradiated yttrium metal target using hydroxamate column

There are many different methods which can be used to separate small amounts (GBq or mCi or ≈10^–5^ mol·L^−1^) of ^88^Zr or ^89^Zr from gram amounts of yttrium. Many separation methods are available due to the fundamental difference of Zr^4+^ and Y^3+^ ions and their behavior in aqueous solutions. According to Shannon^53^, effective ionic radii of Zr^4+^ and Y^3+^ in eightfold coordination are equal to 0.84 Å and 1.019 Å, respectively. Generally, the aqueous solution chemistry of Y is similar to the solution chemistry of heavy lanthanides, while the solution chemistry of Zr is similar to Hf and is complex due to high charge and small ionic radius. Even at trace concentrations (10^–9^ to 10^–11^ mol·L^−1^), Zr^4+^ is easily hydrolyzed and complexed in aqueous media and tends to form various polymeric species and colloids even in acidic HCl media (≈ pH < 1.5). The aqueous chemistry of Zr^4+^ is further complicated by slow polymerization kinetics. Hydrolysis of Zr has been studied by many authors and a comprehensive review is available^54^. According to the review^54^ Zr^4+^ can form various monomeric and polymeric species and the experimental data show that Zr^4+^ starts to relatively slowly hydrolyze at concentrations of H^+^ of 0.1 mol·L^−1^ in NaClO_4_ media. Therefore, it was concluded that a concentration of HCl of ≈2 mol·L^−1^ is enough to avoid Zr^4+^ polymerization and keep it mostly as a free Zr^4+^ ion or as zirconium chloride complex.

Some of the separation methods of trace amounts of Zr from gram amounts of Y include:

1. Extraction chromatography of Zr via hydroxamate-based resins or other resins
2. Anion exchange chromatography of Zr
3. Cation exchange chromatography of Zr

A literature review on previous efforts shows that the separation of small amounts of ^89^Zr or ^88^Zr from grams of Y via extraction chromatography with the hydroxamate-based resin is the most widely used method^5,19,44,45,47,49^. The separation method was developed by Mejs et al.^44^ and studied in more details by Verel et al.^49^ and Holland et al.^19^ and is based on the ability of Zr^4+^ to form strong complexes with hydroxamates over a wide range of HCl and HNO_3_ concentrations. The method results in a radiopharmaceutical grade, high purity ^89^Zr. The stability constants of Zr aqueous complexes with various hydroxamate ligands are very high (log_10_(*β*^*0*^) ≈ 40)^55,56^. Trace amounts of Zr^4+^ are very efficiently retained on the hydroxamate resin in HCl or HNO_3_ media (D-values range from 10^4^ to 10^5^) while the affinity of Y^3+^ is limited (D-values range from 1 to 10)^44^. As discussed in the targetry part of this work, typical impurities in the Y metal target might include other rare-earth metals. The affinity of lanthanides and Sc for hydroxamate functional groups is expected to be similar to Y and thus, the method can be also used to separate Zr from other rare-earth metals. Other possible impurities in Y metal include Ca, Fe and Ta. Titanium could also be present as was shown in this and other works^44^ (^48^V was measured and produced via (p,n) reaction on natural ^48^Ti). The affinity of Ca to hydroxamate resin is expected to be limited over the whole range of HCl concentrations and the retention of Fe is limited from 1 to 6 mol·L^−1^. D-values of some tetra- and pentavalent metals including Ti and Ta on hydroxamate-based resin are very high over a wide range of HCl concentrations^57^ and these elements will follow Zr in the separation procedure. Elution of Zr from hydroxamate resin can be performed using various chelating agents, but usually oxalic acid is used because ^89^Zr oxalic acid eluate can be used directly to rapidly and efficiently label monoclonal antibodies^49^. Zirconium’s affinity for hydroxamate functional groups is very high and usually ≈10 mL of 1 mol·L^−1^ oxalic acid (which is close to saturation) is used for elution. The decimal logarithm of stability constant of the predominant [Zr(C_2_O_4_)_4_]^4−^ complex at zero ionic strength (log_10_ *β*^0^) is 29.7^58^, while the stability of Ti and some other elements is lower and some degree of separation from Zr can be achieved. Hydroxamate-based resin can be easily synthesized by functionalizing a weak cation exchange resin with hydroxamate groups using the previously described procedure^19^ and is commercially available. Other extraction chromatographic resins (TEVA, UTEVA, TRU and LN resins) can also be used to separate ^89^Zr from irradiated Y targets^51^.

Another widely used method for the separation of trace Zr from bulk Y is an anion exchange chromatography^9,50,59^ and strong anion exchange resins based on quaternary ammonium functional groups are well-established. The method is based on the formation of ZrCl_5_^−^ and ZrCl_6_^2−^ complexes at HCl concentrations of 8 mol·L^−1^ or higher, while yttrium does not form negatively charged complexes in HCl media. The method has similar selectivity as the hydroxamate method, because usually only tetra- or pentavalent metals form negatively charged complexes in concentrated HCl. Many anion exchange resins are commercially available and O’Hara and co-workers^59^ evaluated the performance of three different anion exchange resins for the isolation of ^89^Zr: AG 1-X10, AG MP-1 M and Toyopearl QAE-550C. All three resins are quaternary ammonium based. The authors recommend using AG MP-1 M due to its increased ability to retain ^89^Zr from solutions bearing high dissolved Y concentrations. Elution of ^89^Zr from an anion exchange column can be performed using pure HCl with concentrations of 6 mol·L^−1^ or less as well as some other mineral acids, but addition of some F^-^ to the HCl improves Zr elution profiles and its elution selectivity. This is probably due to the formation of mixed Zr complexes with both Cl^−^ and F^−^. In general, selective elution of Zr from an anion exchange column can be performed, and separation of many other elements (e.g. Nb, Ta, W, Mo)^60^ can be obtained.

Strong cation exchange resins can be also used to separate Zr from many elements, including Hf, by selective Zr elution. This method was used by some authors^43,50^ for the separation of ^88^Zr and ^89^Zr from irradiated Y metal and Y_2_O_3_ targets. To the best of our knowledge, separation of ^88^Zr or ^89^Zr from irradiated Y targets using radiation-resistant inorganic sorbents (Al_2_O_3_, TiO_2_ etc) has not been developed yet.

**Figure 2:**
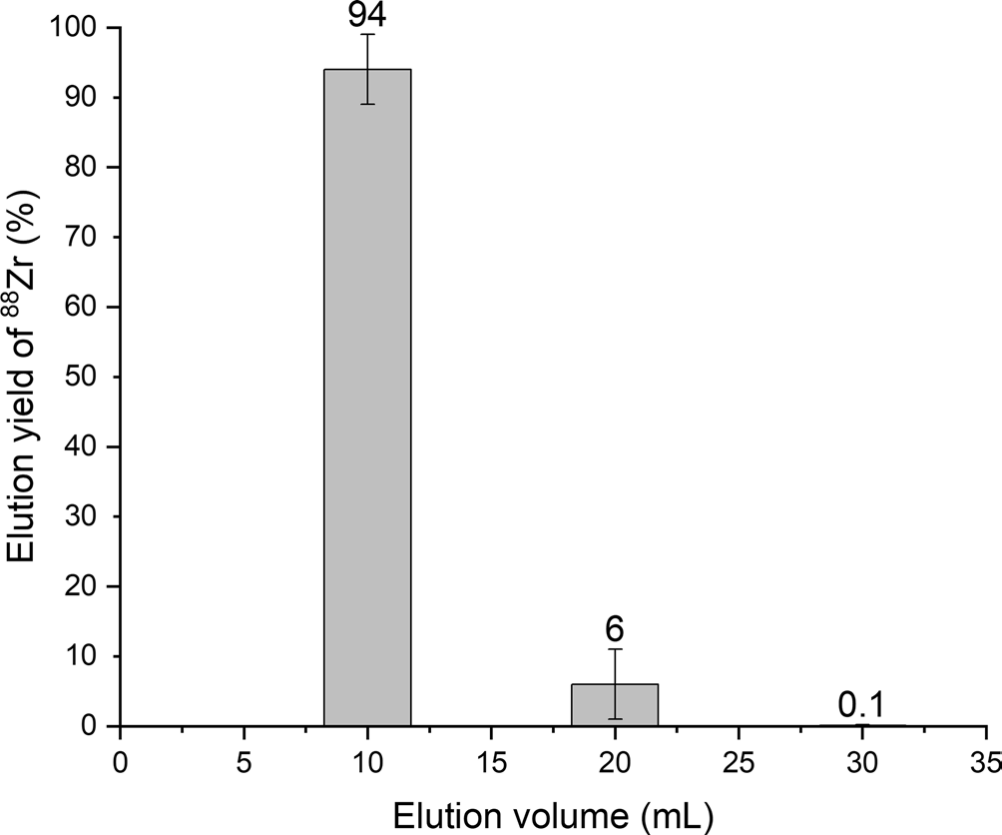
Elution profile for ^88^Zr on a hydroxamate column (2 g). Error bars correspond to 1σ standard deviation calculated from duplicates.

In this work, hydroxamate resin was selected for the separation of ^88^Zr and the method was scaled up to separate tens to hundreds GBq amounts of ^88^Zr (≈10^–5^ mol·L^−1^) from ≈20 g of irradiated Y metal targets. The Y targets were dissolved in 150 mL of 6 mol·L^–1^ HCl and subsequently diluted to 450 mL with water to attain a final concentration of 2 mol·L^−1^. The target solution contained GBq amounts of ^88^Zr, as well as other co-produced radionuclides. This solution was loaded on the custom, in-house made column filled with 2 g of hydroxamate resin and pre-washed first with water and then with 2 mol·L^−1^ HCl. This eluate was collected in 50 mL portions and measured via HPGe detector. The main gamma emission of ^88^Zr (392.1 keV) was not present in these spectra which means that ^88^Zr breakthrough did not occur and more than 99% of ^88^Zr was loaded. After that, the column was washed with ≈50 mL of 2 mol·L^−1^ HCl to remove any possible Y residues. Zirconium-88 was eluted via three 10 mL portions of 1 mol·L^−1^ C_2_H_2_O_4_ and its elution profile is shown in Fig. 2.

As shown above, 94(5)% of the ^88^Zr was eluted in the first portion of 10 mL of 1 mol·L^−1^ C_2_H_2_O_4_, while the second and third 10 mL elutions resulted in 6(5)% elution and 0.1(1)% elution of ^88^Zr, respectively. Similar ^89^Zr elution yields from hydroxamate columns were previously reported^45,49^. Only the first elution was used for further experiments and the second and third elutions were discarded. Only gamma emissions of ^88^Zr, ^89^Zr and weak gamma emissions of ingrown ^88^Y were present in the measured spectrum, which means that ^88^Zr was efficiently separated from stable Y as well as the ^48^V impurity with separation factors of more than 300.

For the first elution (10 mL of 1 mol·L^−1^ C_2_H_2_O_4_ with 94(5)% of ^88^Zr), the oxalic acid was decomposed by the addition of 15 mL of 15.5 mol·L^−1^ HNO_3_^20,21^:

$${\text{3H}}_{{2}} {\text{C}}_{{2}} {\text{O}}_{{4}} \, + \,{\text{2HNO}}_{{3}} \, \rightleftharpoons \,{\text{6CO}}_{{2}} \, + \,{\text{2NO}}\, + \,{\text{4H}}_{{2}} {\text{O}}.$$

This procedure was repeated 2 more times, then the sample was evaporated again and ≈10 mL of 6 mol·L^−1^ HCl added. This sample was diluted and measured via ICP-OES. Results of the ICP-OES measurements are listed in Table 4.

**Table 4:** Elements measured in the ^88^Zr sample obtained from Target 1.

Element	Wavelength (nm)	Concentration (µg·L^−1^)
Zr	339.197	20.5(1)
Y	371.029	< 5
Ca	396.847	8.3(3)
Fe	259.939	< 5
Ti	334.94	< 5
Ta	240.063	< 50
Al	396.153	< 20

Uncertainties in brackets are 1σ standard deviations.

As shown in Table 4, Ca was measured in the ^88^Zr sample. Most probably Ca was present in mineral acids used (HCl and HNO_3_) and was concentrated during many evaporations. Concentration of the measured Zr was quite low due to significant dilution of the aliquot taken from a highly radioactive ^88^Zr sample. Preliminary experiments with the stable, cold Zr without addition of any radiotracers did not require dilutions or evaporations and the measured concentration of Ca was below the detection limit (≈5 µg·L^−1^) however, some amount of stable Y was detected. Detection of Y showed that additional column washes with 2 mol·L^−1^ HCl are required to completely remove all the Y. Stable Ca isotopes and stable monoisotopic ^89^Y are nearly transparent for neutrons and therefore the presence of these elements would not affect neutron transmission measurements. Results obtained in this work can be compared to the result obtained by Queern and co-workers^45^ who used ICP-MS to measure stable impurities in the ^89^Zr sample, which was produced via irradiation of various Y metal targets, and also detected some stable Y together with Zr, Al, and Fe.

### Preparation of the ^88^Zr target for neutron transmission measurements

Recently, a new instrument for neutron transmission measurements was designed and commissioned at LANSCE. This instrument (DICER) was developed to study total neutron and capture cross sections and is especially useful to measure total neutron cross sections of highly radioactive samples, like ^88^Zr, as previously discussed.

Given the extremely large thermal capture cross section of ^88^Zr of 8.61·10^5^ b^9^, the main goal of the DICER experiment was to quantify the resonance presumably responsible for such a large cross section. Considering the large neutron capture cross section and the small 1 mm diameter neutron beam available at the sample position, only a small amount (~1.4 µg) of ^88^Zr was estimated to be needed. Simulations with the *R-*matrix code SAMMY^61^ indicated that this amount of ^88^Zr should be sufficient for accurately determining the parameters of the resonance responsible for the large thermal cross section.

To obtain the total neutron cross section for the nuclide of interest (e.g., ^88^Zr), two transmission measurements are needed: sample in and sample out. Then, the ratio of these two measurements, normalized to the neutron flux in each measurement, is the transmission for the nuclide of interest, from which the total neutron cross section can be calculated. In the present case, the sample for the sample-in measurement contains ^88^Zr itself, the sample matrix, and any “windows” used to contain the sample through which the neutron beam must pass. The sample for the sample-out measurement then contains the same materials as the sample in, except the ^88^Zr. Hence, the sample matrix and “windows” can be made from many materials, provided that they do not absorb or scatter so many neutrons that the measurement takes too long time.

The sample must also be uniform to ensure that the extracted resonance parameters are accurate. Uniform atom distribution can be achieved in gaseous, liquid and solid forms. However, experimental work with volatile radioactive compounds requires enormous radiation protection precautions. Chemical synthesis of simple inorganic solid Zr compounds usually results in powder and experimental work with radioactive powder is also challenging. Moreover, filling of the experimental container with radioactive ^88^Zr powder and packing this powder to this container to achieve uniform distribution is difficult. Special techniques have to be used to obtain uniform distribution of ^88^Zr atoms in solid form e.g., electroplating, inject printing or others. Metal ions undergo various interactions in solution including electrostatic and are usually equally distributed in the liquid form as charged ions or complexes. Moreover, working with highly radioactive liquids is easier from the radiation protection point of view. The required amount of ^88^Zr was small enough (~1.4 µg) that it is feasible to dissolve it in liquid solvent within the available volume (~10 µL sample, assuming a 1.2-mm-diameter and 1 cm long sample container). This approach should ensure a uniform sample and greatly facilitate loading the material into a suitable, small container via, for example, a µL syringe. Therefore, a liquid ^88^Zr sample is the most suitable form for neutron transmission measurements. It can be summarized that there are a few important requirements for the ^88^Zr sample prepared for neutron transmission measurements at DICER:

- A sufficient amount of ^88^Zr for accurately determining the parameters of the resonance responsible for the large thermal cross section
- Neutron transparent sample matrix and minimization of neutron scatterers such as hydrogen
- Uniform distribution of ^88^Zr atoms in the sample matrix and container
- The sample should be compatible with DICER’s form factor (1.2 mm in diameter, 1 cm in length).

Various solvents can be used to dissolve and prepare ~1.4 µg of ^88^Zr sample. Because hydrogen has a fairly large total neutron cross section at low energies^62^, solvents containing it should be avoided. In contrast, the total neutron cross section for deuterium is only about 1/7 of that of hydrogen at these energies, so deuterated solvents are acceptable. For example, neutron transmission at thermal energy for a water sample of 10 µL volume and 1.2 mm diameter is only about 17%, whereas for deuterated water of the same dimensions it is about 78%. Carbon tetrachloride is the most common organic solvent which does not contain H atoms and also has acceptably high neutron transmission in the present case. It is a non-polar solvent which is liquid at room temperature and is not flammable. It cannot be used on its own to dissolve enough ^88^Zr (1.4 µg in 10 µL) but can be used as a diluent in the solvent extraction system. It means that various organic molecules (extracting agents) can be dissolved in CCl_4_ and this extraction system can be used to extract ^88^Zr from the aqueous phase into the CCl_4_ phase. Most of the extracting agents are organic molecules which means that they also contain H atoms, but their concentration can be kept relatively low (e.g. 0.1 mol·L^−1^). A comprehensive review of the solvent extraction systems for the Zr extraction is available^63^ and shows that many extracting agents can extract Zr from aqueous nitrate or chloride media into CCl_4_. In this work two common solvent extraction systems were selected and compared: tributyl phosphate (TBP) in CCl_4_ and 2-thenoyltrifluoroacetone (TTA) in CCl_4_.

#### Solvent extraction system for ^88^Zr target: tributyl phosphate or 2-thenoyltrifluoroacetone in carbon tetrachloride

2-thenoyltrifluoroacetone (TTA) is a dicarbonyl molecule (C_8_H_5_F_3_O_2_S) which can be used for extraction of various metal ions including Zr. A zirconium ion combines with the enol form of TTA. Tributyl phosphate (TBP) is an ester of phosphoric acid with *n*-butanol (PO(C_4_H_9_O)_3_) and is widely used in solvent extraction, especially for the extraction of lanthanides and actinides from aqueous nitrate media. Both TTA and TBP molecules contain H atoms and as discussed before, H atoms have high total neutron cross section and limit neutron transmission. Therefore, it is important to calculate number of H and other atoms in each extraction system and then evaluate the neutron transmission through the sample.

Solvent extraction of Zr from nitrate media into an organic phase containing TTA (C_8_H_5_F_3_O_2_S) as extracting agent and carbon tetrachloride (CCl_4_) as a diluent was studied by El-Hefny and co-workers^64^ and it was shown that extraction of Zr from aqueous nitrate media occurs via the following ion exchange mechanism:

$${\text{Zr}}^{{{4} + }} + {\text{ NO}}_{{3}}^{ - } + {\text{3HTTA}}_{{({\text{aq}})}} = {\text{ ZrNO}}_{{3}} \left( {{\text{TTA}}} \right)_{{{3 }({\text{org}})}} + {\text{ 3H}}^{ + }$$

Combining the extraction equilibrium constant (K_ex_) for the reaction above and Zr distribution ratio (D) results in:

$$D=\frac{{K}_{ex}\cdot {{[NO}_{3}]}^{-}\cdot {[HTTA]}_{org}^{3}}{{{[H}^{+}]}^{3}}$$

The extraction equilibrium constant for the reaction above is 99.73 and ≈1 mol·L^−1^ is optimal concentration of HNO_3_ for Zr extraction^64^.

TBP extracts zirconium nitrate complex via solvating mechanism^65^:

$${\text{Zr}}^{{{4} + }} + {\text{ 4NO}}_{{3}}^{ - } + {2}\left( {{\text{TBP}}} \right)_{{({\text{aq}})}} \to \, \left[ {{\text{Zr}}\left( {{\text{NO}}_{{3}} } \right)_{{4}} \left( {{\text{TBP}}} \right)_{{2}} } \right]_{{({\text{org}})}}$$

The D-value for this reaction is equal to:

$$D={K}_{ex}\cdot {[{NO}_{3}^{-}]}^{4}\cdot {[TBP]}_{org}^{2}$$

As shown in the equation above, the D-value and amount of extracted Zr significantly depends on the concentration of TBP and HNO_3_. According to the literature^65^ *K*_*ex*_ for the reaction above is 0.016 at ≈5 mol·L^−1^ of HNO_3_.

According to definition the percentage of extraction is:

$$\%E=\frac{100\cdot D}{1+D}$$

**Figure 3:**
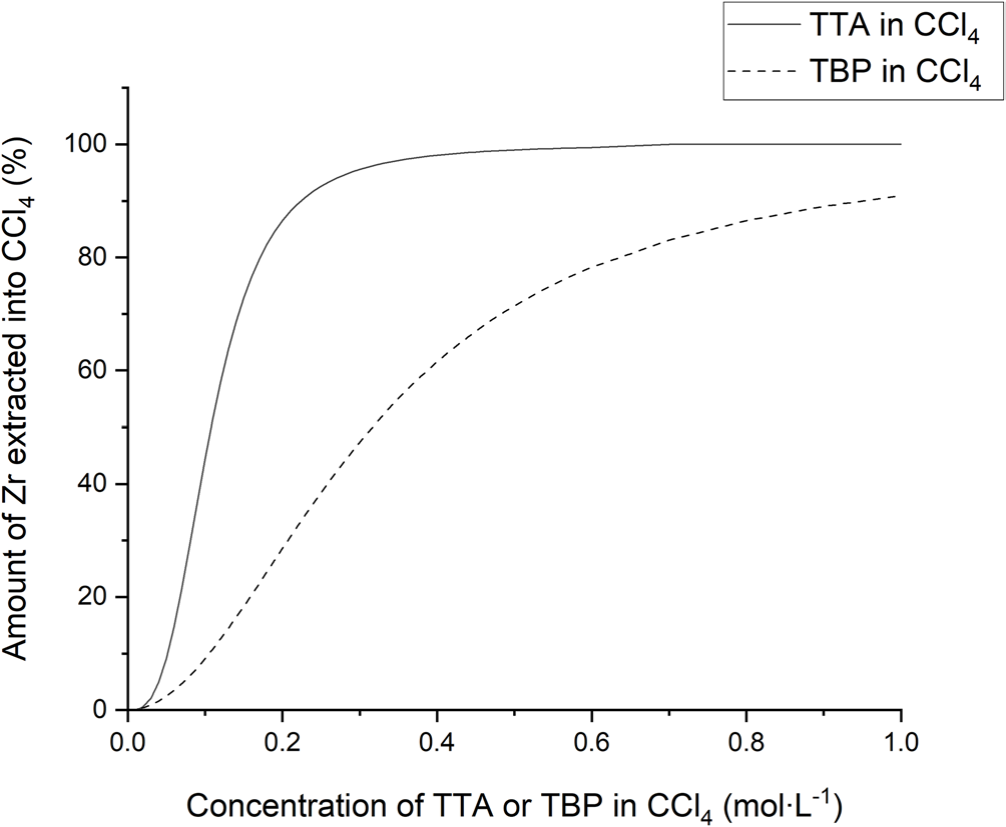
Amount of Zr extracted into CCl_4_ as a function of TTA or TBP concentration in CCl_4_ from 1 mol·L^−1^ HNO_3_ (TTA) and 5 mol·L^−1^ HNO_3_ (TBP).

The amount of Zr extracted into CCl_4_ as a function of TTA or TBP concentration in CCl_4_ can be calculated by substituting the percentage of extraction into D-values and is shown in Fig. 3.

As shown in Fig. 3, 0.3 mol·L^−1^ of TTA results in 95.6% of Zr extracted from 1 mol·L^−1^ HNO_3_ into CCl_4_, while the same concentration of TBP gives only 47.4% of Zr extracted from 5 mol·L^−1^ HNO_3_ into CCl_4_. Moreover, one TTA molecule contains only five H atoms and one TBP molecule contains twenty-seven H atoms. Therefore, it can be concluded that an ^88^Zr sample prepared using TTA in the CCl_4_ system contains less H atoms and has higher neutron transmission.

**Figure 4:**
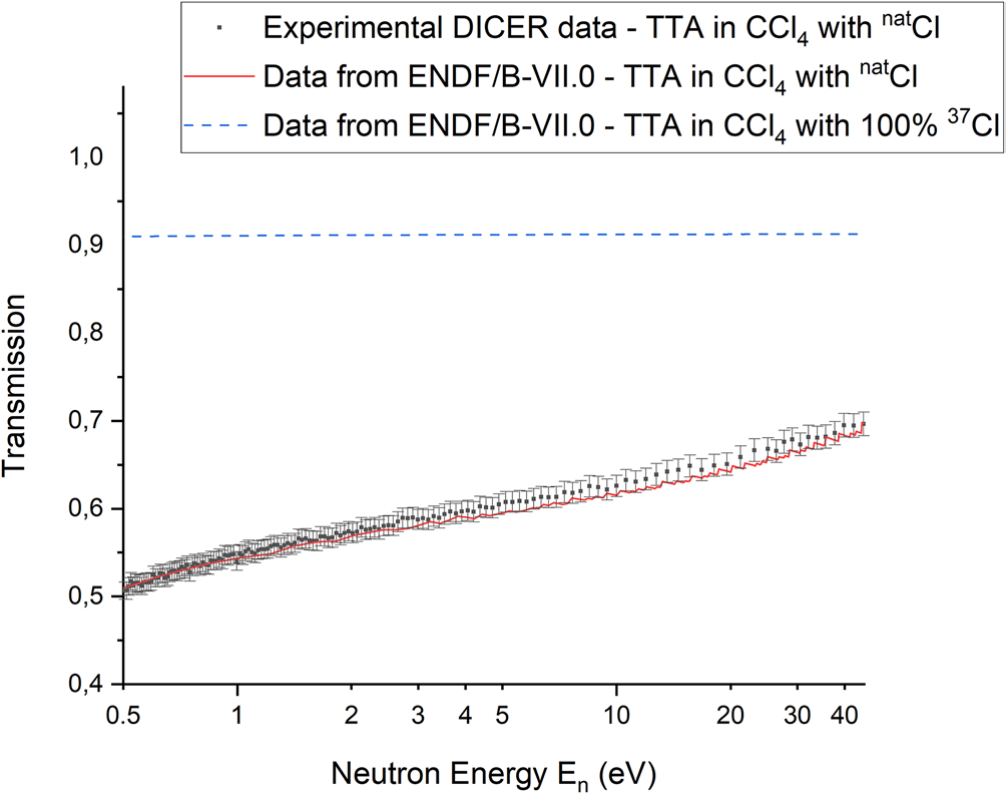
Measured DICER and ENDF neutron transmission through TTA/CCl_4_^nat^Zr target: 8 µL of CCl_4_ sample with 0.3 mol·L^−1^ TTA with 1.4 µg of ^nat^Zr and Pb windows. Error bars correspond to 1σ.

Neutron transmission measured through a blank ^nat^Zr target prepared using TTA in the CCl_4_ solvent extraction system and Pb windows is compared to the expected transmission using the latest evaluated cross sections^62^ in Fig. 4.

As shown in Fig. 4, neutron transmission though the blank ^nat^Zr target prepared using TTA in CCl_4_ solvent extraction system and Pb windows is acceptably high in the energy range shown and experimental DICER data are in a good agreement with the Evaluated Nuclear Data File (ENDF). Thus, Fig. 4 illustrates that ^nat^Zr blank TTA/CCl_4_ sample performed as expected and that neutron transmission through this sample is high enough to see expected attenuation with the ^88^Zr sample. The transmission measurements were made with a cadmium filter in the beam which eliminates neutrons below about 0.3 eV and therefore measurements at thermal energy (0.0253 eV) were not possible. However, the calculated transmission at thermal energy, using the latest evaluation, is lower than desired, indicating that the alternative heavy water matrix is preferred for the ^88^Zr experiment.

#### Heavy water system for ^88^Zr target: deuterium chloride in deuterium oxide

**Figure 5:**
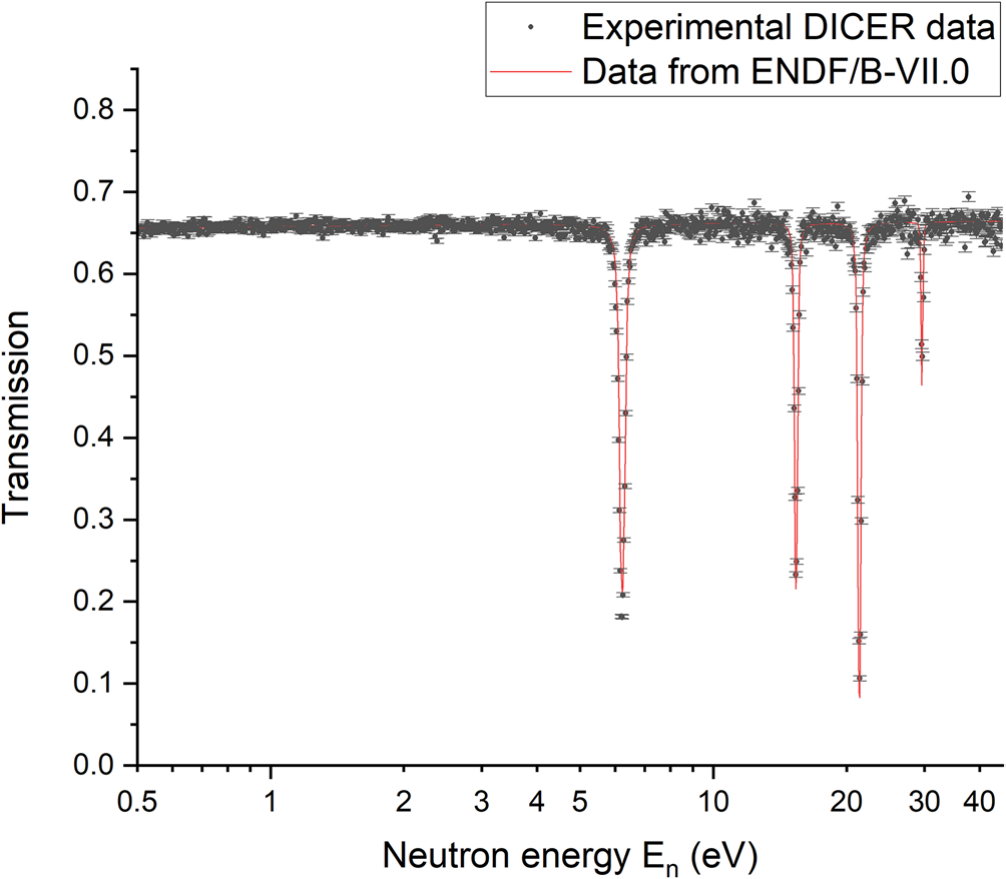
Measured DICER and ENDF neutron transmission through DCl/D_2_O ^nat^Zr target: 8 µL of 2 mol·L^−1^ DCl in D_2_O (with 99.95% D atoms) and 1.4 µg of ^nat^Zr and Pb windows. Error bars correspond to 1σ.

Heavy water (D_2_O) is another hydrogen-free solvent which can be used to prepare an ^88^Zr target and various deuterium compounds including D_2_O and some mineral deuterium acids (e.g., DCl, DNO_3_) with deuteration degrees of as much as 99.95% are commercially available. Pure heavy water can be used to prepare the target, but as discussed above, Zr^4+^ is easily hydrolyzed and polymerized in aqueous media and similar behavior can be expected in pure heavy water at neutral pH. Formation of polymeric Zr species or sorption of Zr could result in a non-uniform distribution of Zr atoms in the target and compromise neutron transmission measurements. Therefore, the ^88^Zr target should be prepared using deuterium-based acid in D_2_O. A concentration of DCl of 2 mol·L^−1^ in D_2_O was selected as potential solvent for ^88^Zr target preparation. Neutron transmission through the blank ^nat^Zr target prepared using 2 mol·L^−1^ DCl in D_2_O (with 99.95% D atoms) and Pb windows is shown in Fig. 5.

According to Fig. 5 the neutron transmission through the ^nat^Zr target prepared using 2 mol·L^−1^ DCl in D_2_O is sufficiently high and approximately 35% of neutron flux is absorbed by the sample matrix (2 mol·L^−1^ DCl in D_2_O) and Pb windows. Thus, 2 mol·L^−1^ DCl in D_2_O is a suitable matrix for the ^88^Zr target for neutron transmission measurements at DICER. Four resonances shown in the Fig. 5 are due to 2.6% (by weight) of Sb impurities in the Pb windows.

However, 99.95% deuteration is quite high and deuterium samples with such a high purity should be preferably kept in inert atmosphere because H atoms from the air moisture can be exchanged with the D atoms in such samples and contaminate them. The rate of this exchange is difficult to calculate. Unfortunately, an inert atmosphere cannot be created in the hot cells used for ^88^Zr target preparation, therefore the most reasonable way to verify if H exchange could result in significant H contamination of DCl/D_2_O samples is to prepare and measure blank samples with natural Zr.

From the chemical point of view, the preparation of a 2 mol·L^−1^ DCl ^88^Zr target is easier compared to the preparation of a TTA/CCl_4_ sample.

#### Design of the zirconium-88 sample can for DICER

A special can for the ^88^Zr sample needs to be designed and manufactured to perform neutron transmission measurements at DICER. The can should meet the following requirements:

1. Any material which will be in the beam should be as neutron transparent as possible
2. Its geometry should be compatible with DICER’s binocular collimator
3. The zirconium-88 sample inside the can (1.2 mm in diameter and 1 cm in length) should fit a neutron beam available at the sample position
4. The can should be hermetically sealed inside the hot cell
5. Can material outside the neutron beam should have a high absorption for gamma rays to reduce dose to personnel loading the sample into DICER.

**Figure 6:**
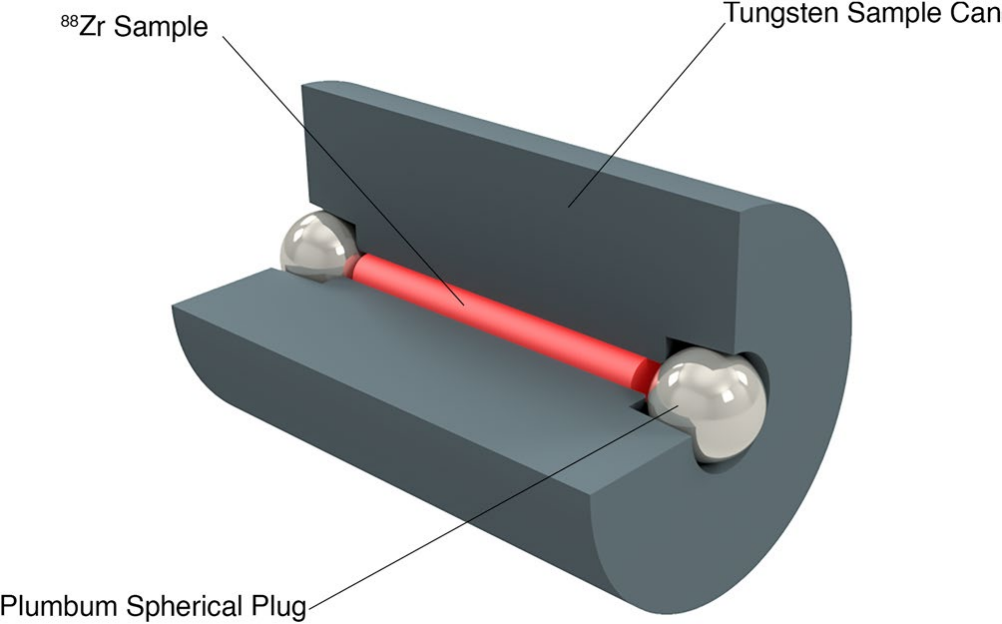
Design of the zirconium-88 sample can used for the neutron transmission measurements at DICER.

The design of the ^88^Zr sample can is shown in Fig. 6.

As shown in Fig. 6, a sample can is made from tungsten with an empty cylindrical space of 1.2 mm in diameter and 1 cm in length (red) to fit the ^88^Zr sample. The whole can is 1 cm in diameter and 1.5 cm long. The hole in the tungsten can was 1.2 mm diameter to allow for some possible alignment error with the 1-mm-diameter collimator. The can can be hermetically sealed by pressing a 2.8 mm diameter lead sphere with a lever press, inside a hot cell. Due to high dose rates, the transfer of the 8 µL sample with ^88^Zr needs to be performed inside the hot cell.

#### Automated station for the zirconium-88 sample transfer

**Figure 7:**
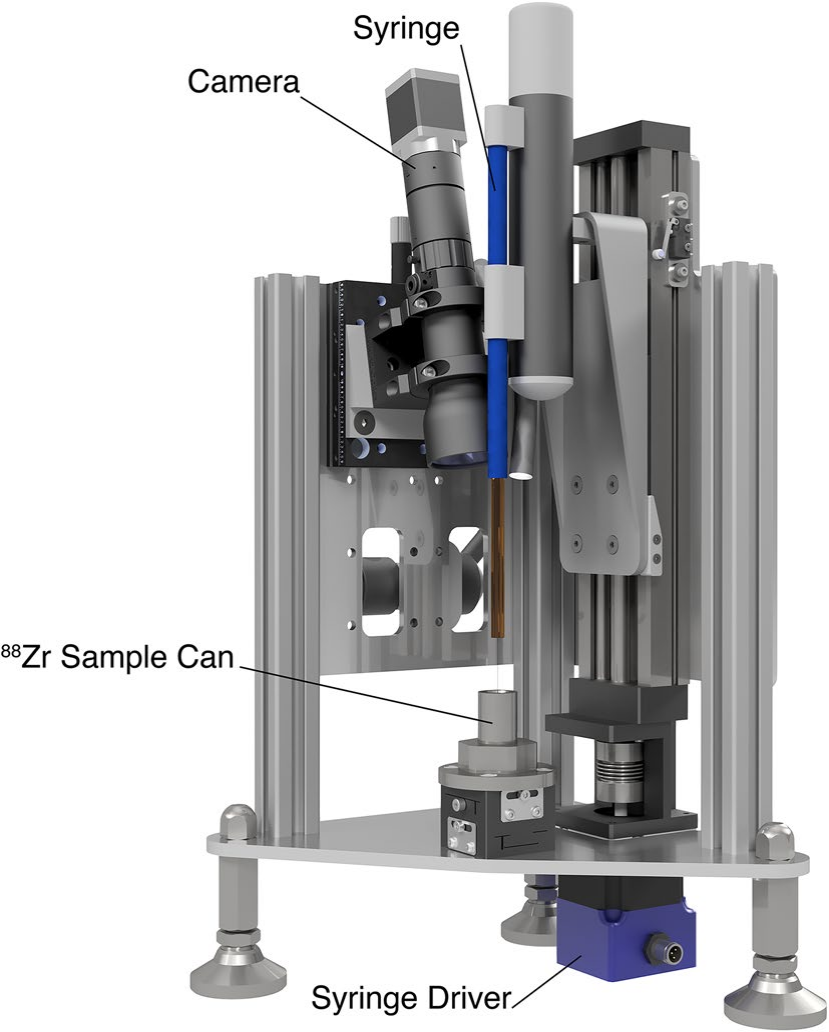
Automated station for transferring of 8 µL sample with ^88^Zr.

The automated station for transferring of 8 µL sample with ^88^Zr from a glass vial to the tungsten can (Fig. 6) is shown in Fig. 7.

The station was installed inside the hot cell and controlled remotely. It consists of a glass syringe (Hamilton) which is driven mechanically by a syringe driver. The station was calibrated outside of the hot cell first. A steel sample holder with a 0.3 mL glass V-vial filled with the 100 µL of ^88^Zr dissolved in 2 mol·L^−1^ DCl from target 2 was placed in the sample position. The glass syringe was moved down, 8 µL of sample was withdrawn and lifted back to the initial position. After that, the glass vial was removed, and the tungsten can sealed on the bottom with a lead sphere was placed in the sample position. The syringe was moved down to fit the 1.2 mm hole and 8 µL of sample was dispensed inside the can (The dispensing is shown in Supplementary video). The can was moved to the lever press and the 2.8 mm diameter Pb sphere plug was then used to seal the top of the can. The can was placed back in the sample position and its seal integrity was checked visually using a camera which the station was equipped with (Fig. S1 and S2 in Supplementary). After seal integrity was ensured, the sample can with the 37 MBq (1 mCi) of ^88^Zr in 8 µL of 2 mol·L^−1^ DCl was taken out of the hot cell and delivered to the LANSCE facility for the neutron transmission measurements. The same experiment with higher ^88^Zr activities from target 3 showed that precipitate can be formed after the matrix swap from HCl to 2 mol·L^−1^ DCl. This indicates that solubility of Zr in DCl is lower than in HCl, therefore 8 µL of 2 mol·L^−1^ HCl was used to prepare ≈1 GBq (27 mCi) of ^88^Zr for the second DICER measurements.

## Conclusion

In this work, a process for the production of ^88^Zr in a proton beam was scaled up from the MBq (mCi) to hundreds of GBq (Ci) amounts. Zirconium-88 was produced via irradiation of ≈20 g yttrium metal targets in a ~16–34 MeV proton beam at a current of 100–200 µA at the LANL Isotope Production Facility. Produced ^88^Zr and ^89^Zr activities were within 1σ standard deviation of the expected activities, predicted using TENDL-2019 cross section data^38^. Activities of co-produced ^88^Y were lower due to the overestimated ^89^Y(p,x)^88^Y cross-section data from TENDL-2019^38^. Nanograms of the produced ^88^Zr and ^89^Zr were separated from ≈20 g of yttrium using a small column filled with 2 g of hydroximate resin and Zr was eluted using 1 mol·L^−1^ oxalic acid with an elution yield of 94(5)% (1σ). Some stable Ca and ^48^V were detected via ICP-OES and HPGe, respectively. Ca is a typical impurity in mineral acids which were used to decompose oxalic acid and to change ^88^Zr sample matrix and ^48^V is activation product of Ti impurity in Y metal target. DCl solution in D_2_O with a deuteration level of 99.95% was selected as the ^88^Zr sample matrix, appropriate for neutron transmission measurements. Deuterium solvent was chosen due to the high neutron transmission of deuterium, as opposed to hydrogen which has lower neutron transmission. Moreover, the liquid form of the ^88^Zr samples ensures an even distribution of ^88^Zr atoms in the sample matrix. An 8 µL sample with ≈3.7 MBq (1 mCi) of ^88^Zr was transferred to a tungsten can with a 1.2 mm diameter hole inside a hot cell using a syringe and automated station, developed specifically for this experiment. Neutron transmission of the obtained ^88^Zr sample was measured at the Device for Indirect Capture Experiments on Radionuclides (DICER). This sample was the first radioactive sample measured at DICER.

## Supplementary Information

Supplementary Video 1.

Supplementary Information 1.

Supplementary Information 2.

## Data Availability

All data generated and analyzed during this study are included in this article (measured concentrations are listed in Tables 3 and 4) and in supplementary information file (neutron transmission measurements are listed in Tables S1 and S2).

## References

[1] Heskamp S (2017). ^89^Zr-immuno-positron emission tomography in oncology: State-of-the-art ^89^Zr radiochemistry. Bioconjug. Chem..

[2] Marquez-Nostra, B. V. & Viola, N. *Radiopharmaceutical Chemistry* (eds. Lewis, J.S., Windhorst, A.D. & Zeglis, B.M.). 371–390 (Springer, 2019).

[3] *Decay Data Evaluation Project (DDEP), Laboratoire National Henri Becquerel, France*. http://www.lnhb.fr/Laraweb/ (2022).

[4] Severin WG, Engle WJ, Barnhart ET, Nickles RJ (2011). ^89^Zr radiochemistry for positron emission tomography. Med. Chem..

[5] Meijs WE (1997). Zirconium-labeled monoclonal antibodies and their distribution in tumor-bearing nude mice. J. Nucl. Med..

[6] Kobayashi H (1999). Evaluation of the in vivo biodistribution of indium-111 and yttrium-88 labeled dendrimer-1B4M-DTPA and its conjugation with anti-tac monoclonal antibody. Bioconjug. Chem..

[7] Chadwick MB (2007). Evaluated iridium, yttrium, and thulium cross sections and integral validation against critical assembly and Bethe sphere measurements. Nucl. Data Sheets.

[8] Prestwood RJ, Thomas KW, Nethaway DR, Smith NL (1984). Measurement of 14-MeV neutron cross sections for ^88^Zr and ^88^Y. Phys. Rev. C.

[9] Shusterman JA (2019). The surprisingly large neutron capture cross-section of ^88^Zr. Nature.

[10] Escher JE (2018). Constraining neutron capture cross sections for unstable nuclei with surrogate reaction data and theory. Phys. Rev. Lett..

[11] Utsunomiya H (2010). γ-ray strength function method and its application to ^107^Pd. Phys. Rev. C.

[12] Utsunomiya H (2013). Photoneutron cross sections for Mo isotopes: A step toward a unified understanding of (γ, n) and (n, γ) reactions. Phys. Rev. C.

[13] Guttormsen M, Ramsøy T, Rekstad J (1987). The first generation of γ-rays from hot nuclei. Nucl. Instrum. Methods Phys. Res. Sect. A.

[14] Guttormsen M, Tveter TS, Bergholt L, Ingebretsen F, Rekstad J (1996). The unfolding of continuum γ-ray spectra. Nucl. Instrum. Methods Phys. Res. Sect. A.

[15] Larsen AC (2011). Analysis of possible systematic errors in the Oslo method. Phys. Rev. C.

[16] Schiller A (2000). Extraction of level density and γ strength function from primary γ spectra. Nucl. Instrum. Methods Phys. Res. Sect. A.

[17] Spyrou A (2014). Novel technique for constraining r-process (n, γ) reaction rates. Phys. Rev. Lett..

[18] O’Brien EM (2020). Novel design and diagnostics improvements for increased production capacity and improved reliability at the Los Alamos isotope production facility. Nucl. Instrum. Methods Phys. Res. Sect. A Acceler. Spectrom. Detect. Assoc. Equip..

[19] Holland JP, Sheh Y, Lewis JS (2009). Standardized methods for the production of high specific-activity zirconium-89. Nucl. Med. Biol..

[20] Kubota M (1982). Decomposition of oxalic acid with nitric acid. J. Radioanal. Chem..

[21] Mason C (2016). The decomposition of oxalic acid in nitric acid. J. Solut. Chem..

[22] Steyn GF (2021). Large-scale production of ^88^Y and ^88^Zr/^88^Y generators: A proof of concept study for a 70 MeV H^−^ cyclotron. Appl. Radiat. Isotopes.

[23] Tárkányi F (2005). Excitation functions for production of ^88^Zr and ^88^Y by proton and deuteron irradiation of Mo, Nb, Zr, and Y. AIP Conf. Proc..

[24] Zaneb H, Hussain M, Amjad N, Qaim SM (2016). Evaluation of nuclear reaction cross section data for the production of ^87^Y and ^88^Y via proton, deuteron and alpha-particle induced transmutations. Appl. Radiat. Isot..

[25] Fassbender M, Jamriska DJ, Hamilton VT, Nortier FM, Phillips DR (2005). Simultaneous ^68^Ge and ^88^Zr recovery from proton irradiated Ga/Nb capsules. J. Radioanal. Nucl. Chem..

[26] Faßbender M (2004). Some nuclear chemical aspects of medical generator nuclide production at the Los Alamos hot cell facility. Radiochim. Acta.

[27] Shusterman JA (2021). Aqueous harvesting of ^88^Zr at a radioactive-ion-beam facility for cross-section measurements. Phys. Rev. C.

[28] Khandaker MU (2012). Investigations of ^89^Y(p, x^)8^6^,8^8^,89^gZr,^86^g^,87^g^,87^m^,88^gY^85^gSr, and ^84^ gRb nuclear processes up to 42 MeV. Nucl. Instrum. Methods Phys. Res. Sect. B.

[29] Levkovski, V. N. *Cross Sections of Medium Mass Nuclide Activation (A=40–100) by Medium Energy Protons and Alpha Particles (E=10–50 MeV)*. (Intervesy, 1991).

[30] Michel R (1997). Cross sections for the production of residual nuclides by low- and medium-energy protons from the target elements C, N, O, Mg, Al, Si, Ca, Ti, V, Mn, Fe Co, Ni, Cu, Sr, Y, Zr, Nb, Ba and Au. Nucl. Instrum. Methods Phys. Res. Sect. B.

[31] Mustafa MG (1988). Measurements and a direct-reaction-plus-Hauser–Feshbach analysis of^89^Y(p, n^)8^9Zr,^8^9Y(p,2n^)8^8Zr, and^8^9Y(p, p^n^^)^88Y reactions up to 40 MeV. Phys. Rev. C.

[32] Omara HM, Hassan KF, Kandil SA, Hegazy FE, Saleh ZA (2009). Proton induced reactions on ^89^Y with particular reference to the production of the medically interesting radionuclide ^89^Zr. Radiochim. Acta.

[33] Saha GB, Porile NT, Yaffe L (1966). (p, xn) and (p, pxn) reactions of yttrium-89 with 5–85-MeV protons. Phys. Rev..

[34] Steyn GF (2011). Excitation functions of proton induced reactions on ^89^Y and ^93^Nb with emphasis on the production of selected radio-zirconiums. J. Korean Phys. Soc..

[35] Uddin MS, Hagiwara M, Baba M, Tarkanyi F, Ditroi F (2005). Experimental studies on excitation functions of the proton-induced activation reactions on yttrium. Appl. Radiat. Isot..

[36] Yang S-C, Song T-Y, Lee Y-O, Kim G (2017). Production cross sections of proton-induced reactions on yttrium. Nucl. Instrum. Methods Phys. Res. Sect. B.

[37] Zhao W, Shen Q, Lu H, Yu W (1992). Investigation of^89^Y(p, n^)8^9Zr^,8^9Y(p,2n^)8^8Zr and^8^9Y(p, p^n^^)^88Y reactions up to 22 MeV. Chin. J. Nucl. Phys..

[38] Koning AJ (2019). TENDL: Complete nuclear data library for innovative nuclear science and technology. Nucl. Data Sheets.

[39] Takács S, Tárkányi F, Sonck M, Hermanne A (2002). Investigation of the^nat^Mo(p, x^)96m^gTc nuclear reaction to monitor proton beams: New measurements and consequences on the earlier reported data. Nucl. Instrum. Methods Phys. Res. Sect. B.

[40] Qaim SM, Sudár S, Scholten B, Koning AJ, Coenen HH (2014). Evaluation of excitation functions of^100^Mo(p, d+pn^)9^9Mo and^10^0Mo (p,2n^)99^mTc reactions: Estimation of long-lived Tc-impurity and its implication on the specific activity of cyclotron-produced^99^mTc. Appl. Radiat. Isot..

[41] Stevenson PC, Nervik WE (1961). The Radiochemistry of the Rare Earths, Scandium, Yttrium, and Actinium.

[42] Dias GM (2018). ^89^Zr for antibody labeling and in vivo studies—A comparison between liquid and solid target production. Nucl. Med. Biol..

[43] Dutta B, Maiti M, Lahiri S (2009). Production of ^88,89^Zr by proton induced activation of ^nat^Y and separation by SLX and LLX. J. Radioanal. Nucl. Chem..

[44] Meijs WE (1994). Production of highly pure no-carrier added ^89^Zr for the labelling of antibodies with a positron emitter. Appl. Radiat. Isot..

[45] Queern SL (2017). Production of Zr-89 using sputtered yttrium coin targets. Nucl. Med. Biol..

[46] Siikanen J (2012). A peristaltic pump driven ^89^Zr separation module. AIP Conf. Proc..

[47] Tang Y (2019). A radiopharmaceutical [^89^Zr]Zr-DFO-nimotuzumab for immunoPET with epidermal growth factor receptor expression in vivo. Nucl. Med. Biol..

[48] Tang Y (2016). A simple and convenient method for production of ^89^Zr with high purity. Appl. Radiat. Isot..

[49] Verel I (2003). ^89^Zr immuno-PET: Comprehensive procedures for the production of ^89^Zr-labeled monoclonal antibodies. J. Nucl. Med..

[50] Kandil SA (2008). A comparative study on the separation of radiozirconium via ion-exchange and solvent extraction techniques, with particular reference to the production of ^88^Zr and ^89^Zr in proton induced reactions on yttrium. J. Radioanal. Nucl. Chem..

[51] Kazakov AG, Aliev RA, Ostapenko VS, Priselkova AB, Kalmykov SN (2018). Separation of ^89^Zr from irradiated yttrium targets by extraction chromatography. J. Radioanal. Nucl. Chem..

[52] Omara HM, Hassan KF, Kandil SA, Hegazy FE, Saleh ZA (2009). Proton induced reactions on ^89^Y with particular reference to the production of the medically interesting radionuclide ^89^Zr. RCA-Radiochim. Acta.

[53] Shannon RD (1976). Revised effective ionic radii and systematic studies of interatomic distances in halides and chalcogenides. Acta Crystallogr. Sect. A Cryst. Phys. Diffract. Theoret. General Crystallogr..

[54] Brown, P. L., Curti, E., Grambow, B. & Ekberg, C. *Chemical Thermodynamics of Zirconium*. Vol. 8 (Elsevier, 2005).

[55] Holland JP (2020). Predicting the thermodynamic stability of zirconium radiotracers. Inorg. Chem..

[56] Toporivska Y (2021). Thermodynamic stability and speciation of Ga(III) and Zr(IV) complexes with high-denticity hydroxamate chelators. Inorg. Chem..

[57] Radchenko V (2017). Proton-induced production and radiochemical isolation of ^44^Ti from scandium metal targets for ^44^Ti/^44^Sc generator development. Nucl. Med. Biol..

[58] Kobayashi T, Sasaki T, Takagi I, Moriyama H (2009). Zirconium solubility in ternary aqueous system of Zr(IV)–OH–carboxylates. J. Nucl. Sci. Technol..

[59] O’Hara MJ, Murray NJ, Carter JC, Morrison SS (2018). Optimized anion exchange column isolation of zirconium-89 (^89^Zr) from yttrium cyclotron target: Method development and implementation on an automated fluidic platform. J. Chromatogr. A.

[60] Bandi WR, Buyok EG, Lewis LL, Melnick LM (1961). Anion exchange separation of zirconium, titanium, niobium, tantalum, tungsten, and molybdenum. Anal. Chem..

[61] Larson, N.M. *Updated Users’ Guide for SAMMY: Multilevel R-Matrix Fits to Neutron Data Using Bayes’ Equations*. Vol. 2 (Oak Ridge National Laboratory, 2008).

[62] Brown, D. A. *et al.* ENDF/B-VIII.0: The 8th major release of the nuclear reaction data library with CIELO-project cross sections, new standards and thermal scattering data. *Nucl. Data Sheets* **148**, 1–142. 10.1016/j.nds.2018.02.001 (2018).

[63] Banda R, Lee MS (2015). Solvent extraction for the separation of Zr and Hf from aqueous solutions. Sep. Purif. Rev..

[64] El-Hefny NE, El-Nadi YA, Daoud JA (2006). Effect of diluents on the extraction of zirconium from nitrate medium by thenoyltrifluoroacetone. Solvent Extr. Ion Exch..

[65] Umezawa H, Hara R (1961). Studies on the extraction of zirconium with organophosphorus compounds. Anal. Chim. Acta.

