# Supplementary material for: Production of zirconium-88 via proton irradiation of metallic yttrium and preparation of target for neutron transmission measurements at DICER
Source: Sci Rep. 2023 Jan 31;13:1736. doi: 10.1038/s41598-023-27993-7 (PMC9889377; doi:10.1038/s41598-023-27993-7)
Supplement: Supplementary file 2 — Supplementary Information 1. [file 41598_2023_27993_MOESM2_ESM.docx]

**Supplementary information**

**Production of zirconium-88 via proton irradiation of metallic yttrium and preparation of target for neutron transmission measurements at DICER**

*Artem V. Matyskin^1,2*^, Athanasios Stamatopoulos^3^, Ellen M. O’Brien^1^, Brad J. DiGiovine^3,4^, Veronika Mocko^1^, Michael E. Fassbender^1^, C. Etienne Vermeulen^1^, Paul E. Koehler^3^*

^1^ Chemistry Division, Los Alamos National Laboratory, P.O. Box 1663, Los Alamos, NM, 87545, USA

^2^ Present affiliation: Radiation Science and Engineering Center, Pennsylvania State University, 135 Breazeale Nuclear Reactor, University Park, PA, 16802, USA

^3^ Physics Division, Los Alamos National Laboratory, P.O. Box 1663, Los Alamos, NM, 87545, USA

^4^ Present affiliation: Q Division, Los Alamos National Laboratory, P.O. Box 1663, Los Alamos, NM, 87545, USA

^*^ Corresponding author: [matyskin.artem@gmail.com](mailto:matyskin.artem@gmail.com)


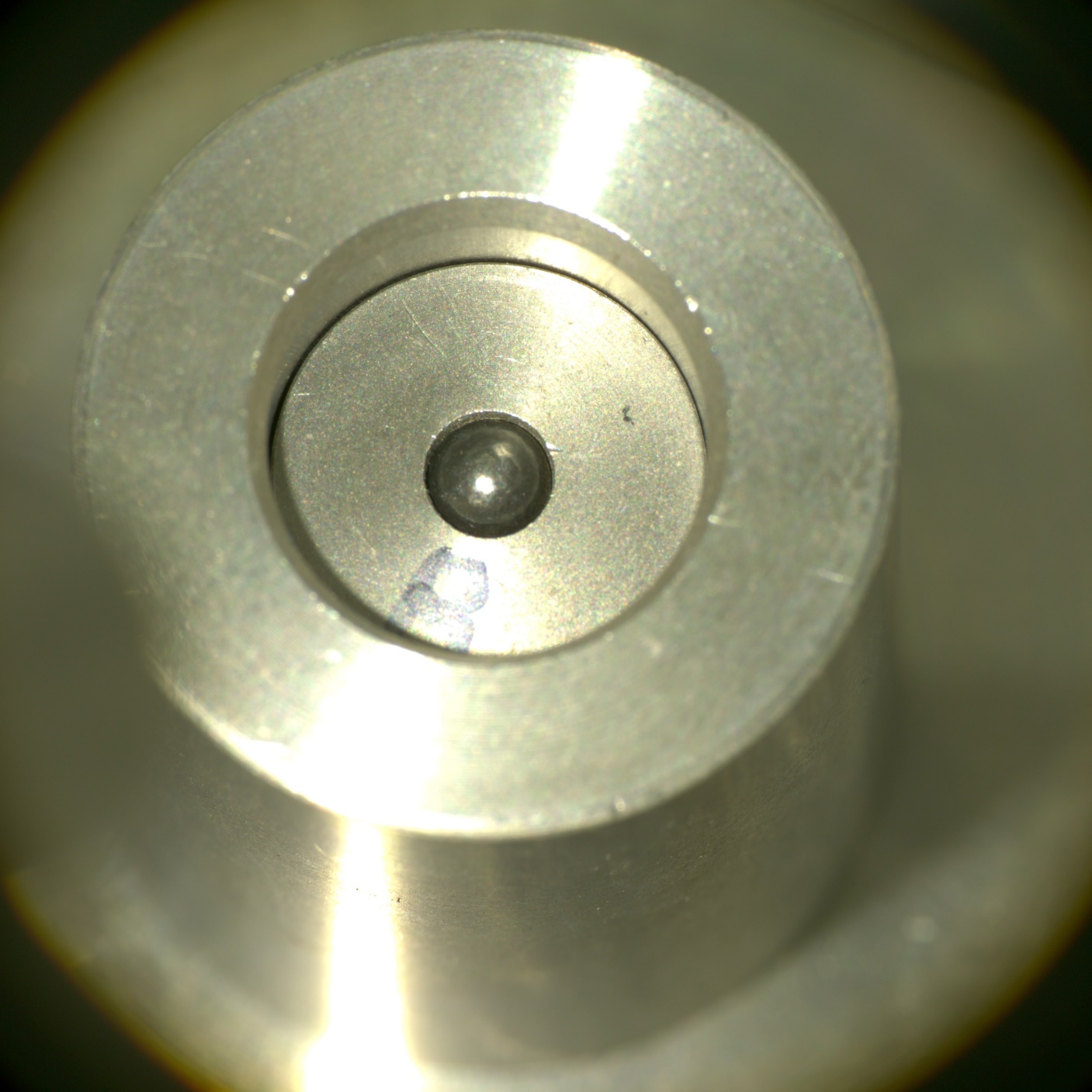


Fig. S1. Lead sphere with 2.8 mm diameter used to seal the sample can containing ^88^Zr sample


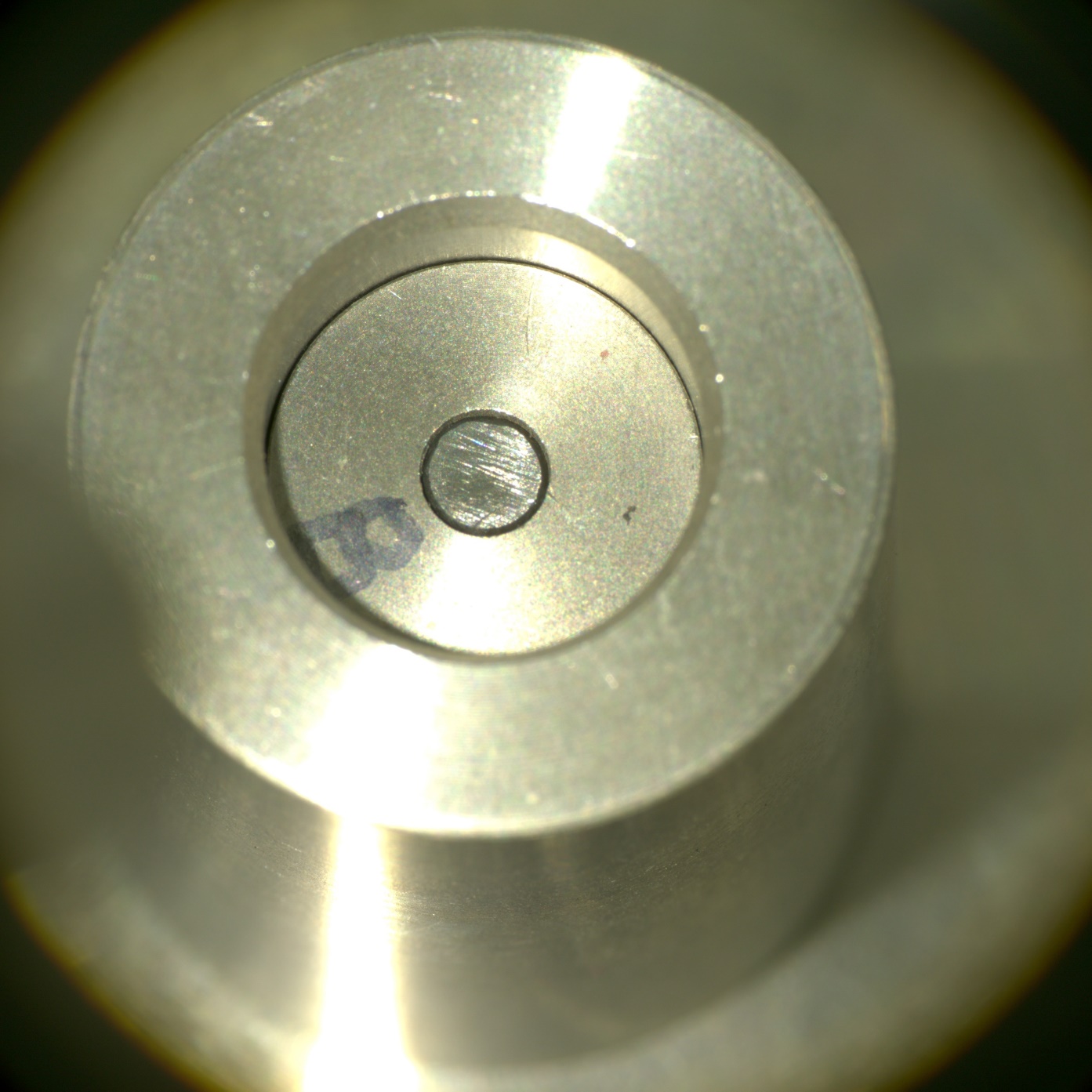


Fig. S2. Hermetically sealed sample can containing ^88^Zr sample, sealed using Pb sphere and lever press inside a hot cell

Table S1. Measured DICER neutron transmission through TTA/CCl_4_ ^nat^Zr target: 8 µL of CCl_4_ sample with 0.3 ·mol·L^−1^ TTA with 1.4 µg of ^nat^Zr and Pb windows

| **Energy (keV)** | **Transmission** | **1σ standard deviation** |
| --- | --- | --- |
| 39.84426 | 0.69489 | 0.01345 |
| 37.66853 | 0.6859 | 0.01328 |
| 35.66626 | 0.68197 | 0.0132 |
| 33.81951 | 0.68057 | 0.01318 |
| 32.1126 | 0.68155 | 0.01319 |
| 30.53171 | 0.67313 | 0.01303 |
| 29.06476 | 0.67877 | 0.01314 |
| 27.70105 | 0.67607 | 0.01309 |
| 26.43111 | 0.66558 | 0.01289 |
| 25.10821 | 0.66783 | 0.01293 |
| 23.11244 | 0.66652 | 0.0129 |
| 21.23056 | 0.65878 | 0.01275 |
| 19.56954 | 0.65057 | 0.01259 |
| 18.09611 | 0.64915 | 0.01257 |
| 16.78304 | 0.64424 | 0.01247 |
| 15.60786 | 0.64851 | 0.01255 |
| 14.55198 | 0.64402 | 0.01247 |
| 13.59971 | 0.64232 | 0.01243 |
| 12.73798 | 0.63885 | 0.01237 |
| 11.95561 | 0.63379 | 0.01227 |
| 11.24319 | 0.63055 | 0.01221 |
| 10.5926 | 0.63239 | 0.01224 |
| 9.99691 | 0.62583 | 0.01211 |
| 9.45006 | 0.62181 | 0.01204 |
| 8.94691 | 0.6246 | 0.01209 |
| 8.48289 | 0.62546 | 0.01211 |
| 8.05406 | 0.61966 | 0.012 |
| 7.65695 | 0.618 | 0.01196 |
| 7.2885 | 0.61885 | 0.01198 |
| 6.94602 | 0.61329 | 0.01187 |
| 6.62712 | 0.61278 | 0.01186 |
| 6.3297 | 0.61322 | 0.01187 |
| 6.05186 | 0.61112 | 0.01183 |
| 5.79191 | 0.60799 | 0.01177 |
| 5.54837 | 0.60878 | 0.01178 |
| 5.31987 | 0.60756 | 0.01176 |
| 5.10519 | 0.60755 | 0.01176 |
| 4.90326 | 0.60501 | 0.01171 |
| 4.71308 | 0.60088 | 0.01163 |
| 4.53375 | 0.60128 | 0.01164 |
| 4.36446 | 0.60241 | 0.01166 |
| 4.20448 | 0.59628 | 0.01154 |
| 4.05314 | 0.598 | 0.01158 |
| 3.90983 | 0.59659 | 0.01155 |
| 3.77398 | 0.59456 | 0.01151 |
| 3.6451 | 0.59678 | 0.01155 |
| 3.5227 | 0.59354 | 0.01149 |
| 3.40637 | 0.58942 | 0.01141 |
| 3.29571 | 0.59142 | 0.01145 |
| 3.19035 | 0.58745 | 0.01137 |
| 3.08997 | 0.58891 | 0.0114 |
| 2.99425 | 0.58758 | 0.01137 |
| 2.90291 | 0.58994 | 0.01142 |
| 2.81568 | 0.58905 | 0.0114 |
| 2.73234 | 0.58935 | 0.01141 |
| 2.65263 | 0.58511 | 0.01133 |
| 2.57637 | 0.58051 | 0.01124 |
| 2.50334 | 0.58107 | 0.01125 |
| 2.43338 | 0.58016 | 0.01123 |
| 2.36631 | 0.57646 | 0.01116 |
| 2.30198 | 0.5787 | 0.0112 |
| 2.24023 | 0.57688 | 0.01117 |
| 2.18094 | 0.57604 | 0.01115 |
| 2.12397 | 0.57864 | 0.0112 |
| 2.0692 | 0.57363 | 0.0111 |
| 2.01652 | 0.57208 | 0.01107 |
| 1.96583 | 0.57423 | 0.01112 |
| 1.91703 | 0.57196 | 0.01107 |
| 1.87002 | 0.56957 | 0.01103 |
| 1.82472 | 0.57223 | 0.01108 |
| 1.78105 | 0.56705 | 0.01098 |
| 1.73893 | 0.56644 | 0.01096 |
| 1.69828 | 0.56835 | 0.011 |
| 1.65904 | 0.56831 | 0.011 |
| 1.62115 | 0.56316 | 0.0109 |
| 1.58454 | 0.56343 | 0.01091 |
| 1.54916 | 0.56384 | 0.01091 |
| 1.51495 | 0.56612 | 0.01096 |
| 1.48185 | 0.56454 | 0.01093 |
| 1.44984 | 0.5658 | 0.01095 |
| 1.41884 | 0.56096 | 0.01086 |
| 1.38884 | 0.55863 | 0.01081 |
| 1.35977 | 0.5607 | 0.01085 |
| 1.3316 | 0.55841 | 0.01081 |
| 1.30431 | 0.55556 | 0.01075 |
| 1.27784 | 0.55868 | 0.01081 |
| 1.25217 | 0.55874 | 0.01082 |
| 1.22727 | 0.55701 | 0.01078 |
| 1.2031 | 0.55454 | 0.01073 |
| 1.17964 | 0.5538 | 0.01072 |
| 1.15686 | 0.55349 | 0.01071 |
| 1.13473 | 0.55261 | 0.0107 |
| 1.11323 | 0.54881 | 0.01062 |
| 1.09234 | 0.55004 | 0.01065 |
| 1.07203 | 0.55348 | 0.01071 |
| 1.05228 | 0.55139 | 0.01067 |
| 1.03307 | 0.54676 | 0.01058 |
| 1.01439 | 0.54831 | 0.01061 |
| 0.9962 | 0.54033 | 0.01046 |
| 0.9785 | 0.54776 | 0.0106 |
| 0.96127 | 0.54754 | 0.0106 |
| 0.94448 | 0.5463 | 0.01057 |
| 0.92814 | 0.54718 | 0.01059 |
| 0.91221 | 0.54185 | 0.01049 |
| 0.89669 | 0.54344 | 0.01052 |
| 0.88157 | 0.54074 | 0.01047 |
| 0.86682 | 0.54057 | 0.01046 |
| 0.85244 | 0.54096 | 0.01047 |
| 0.83842 | 0.53569 | 0.01037 |
| 0.82474 | 0.53643 | 0.01038 |
| 0.81139 | 0.53863 | 0.01043 |
| 0.79836 | 0.53426 | 0.01034 |
| 0.78564 | 0.53583 | 0.01037 |
| 0.77323 | 0.53741 | 0.0104 |
| 0.7611 | 0.53384 | 0.01033 |
| 0.74926 | 0.5277 | 0.01022 |
| 0.7377 | 0.53541 | 0.01036 |
| 0.7264 | 0.53128 | 0.01029 |
| 0.71536 | 0.53348 | 0.01033 |
| 0.70456 | 0.53043 | 0.01027 |
| 0.69401 | 0.52791 | 0.01022 |
| 0.6837 | 0.52945 | 0.01025 |
| 0.67361 | 0.52855 | 0.01023 |
| 0.66375 | 0.52694 | 0.0102 |
| 0.6541 | 0.5225 | 0.01011 |
| 0.64465 | 0.52085 | 0.01008 |
| 0.63542 | 0.52551 | 0.01017 |
| 0.62638 | 0.52633 | 0.01019 |
| 0.61753 | 0.52135 | 0.01009 |
| 0.60886 | 0.5211 | 0.01009 |
| 0.60038 | 0.52425 | 0.01015 |
| 0.59207 | 0.5171 | 0.01001 |
| 0.58394 | 0.51597 | 0.00999 |
| 0.57597 | 0.51686 | 0.01001 |
| 0.56816 | 0.51578 | 0.00999 |
| 0.56052 | 0.51191 | 0.00991 |
| 0.55302 | 0.51443 | 0.00996 |
| 0.54567 | 0.51538 | 0.00998 |
| 0.53847 | 0.51529 | 0.00998 |
| 0.53141 | 0.51229 | 0.00992 |
| 0.52449 | 0.51698 | 0.01001 |
| 0.51771 | 0.51126 | 0.0099 |
| 0.51105 | 0.50658 | 0.00981 |

Table S2. Measured DICER neutron transmission through DCl/D_2_O ^nat^Zr target: 8 µL of 2 ·mol·L^−1^ DCl in D_2_O (with 99.95% D atoms) and 1.4 µg of ^nat^Zr and Pb windows

| **Energy (keV)** | **Transmission** | **1σ standard deviation** |
| --- | --- | --- |
| 0.5013 | 0.6541 | 0.0031 |
| 0.5036 | 0.6492 | 0.003 |
| 0.5059 | 0.6541 | 0.003 |
| 0.5082 | 0.6566 | 0.003 |
| 0.5106 | 0.6579 | 0.003 |
| 0.5129 | 0.6538 | 0.003 |
| 0.5153 | 0.6562 | 0.003 |
| 0.5177 | 0.6459 | 0.003 |
| 0.5201 | 0.6534 | 0.003 |
| 0.5225 | 0.6515 | 0.003 |
| 0.5249 | 0.6598 | 0.003 |
| 0.5273 | 0.6627 | 0.003 |
| 0.5297 | 0.6541 | 0.0029 |
| 0.5322 | 0.6562 | 0.003 |
| 0.5346 | 0.6546 | 0.0029 |
| 0.5371 | 0.6474 | 0.0029 |
| 0.5396 | 0.6583 | 0.0029 |
| 0.5421 | 0.6562 | 0.0029 |
| 0.5446 | 0.6508 | 0.0029 |
| 0.5471 | 0.6529 | 0.0029 |
| 0.5496 | 0.6504 | 0.0029 |
| 0.5522 | 0.6593 | 0.0029 |
| 0.5547 | 0.6534 | 0.0029 |
| 0.5573 | 0.6542 | 0.0029 |
| 0.5598 | 0.655 | 0.0029 |
| 0.5624 | 0.6566 | 0.0029 |
| 0.565 | 0.6591 | 0.0029 |
| 0.5676 | 0.658 | 0.0028 |
| 0.5703 | 0.6623 | 0.0029 |
| 0.5729 | 0.6518 | 0.0028 |
| 0.5755 | 0.6625 | 0.0028 |
| 0.5782 | 0.6535 | 0.0028 |
| 0.5809 | 0.6581 | 0.0028 |
| 0.5835 | 0.6566 | 0.0028 |
| 0.5862 | 0.6543 | 0.0028 |
| 0.5889 | 0.6587 | 0.0028 |
| 0.5917 | 0.6509 | 0.0028 |
| 0.5944 | 0.6574 | 0.0028 |
| 0.5971 | 0.6546 | 0.0028 |
| 0.5999 | 0.6607 | 0.0028 |
| 0.6027 | 0.6604 | 0.0028 |
| 0.6054 | 0.6467 | 0.0028 |
| 0.6082 | 0.6506 | 0.0028 |
| 0.611 | 0.6599 | 0.0028 |
| 0.6139 | 0.6492 | 0.0027 |
| 0.6167 | 0.6598 | 0.0028 |
| 0.6196 | 0.6492 | 0.0028 |
| 0.6224 | 0.6594 | 0.0028 |
| 0.6253 | 0.6585 | 0.0028 |
| 0.6282 | 0.6571 | 0.0028 |
| 0.6311 | 0.6537 | 0.0027 |
| 0.634 | 0.657 | 0.0028 |
| 0.6369 | 0.655 | 0.0027 |
| 0.6399 | 0.6502 | 0.0027 |
| 0.6428 | 0.6583 | 0.0028 |
| 0.6458 | 0.6534 | 0.0027 |
| 0.6488 | 0.6576 | 0.0027 |
| 0.6517 | 0.6455 | 0.0027 |
| 0.6548 | 0.6622 | 0.0027 |
| 0.6578 | 0.6557 | 0.0027 |
| 0.6608 | 0.654 | 0.0027 |
| 0.6639 | 0.6537 | 0.0027 |
| 0.6669 | 0.6589 | 0.0027 |
| 0.67 | 0.6664 | 0.0027 |
| 0.6731 | 0.6628 | 0.0027 |
| 0.6762 | 0.6577 | 0.0027 |
| 0.6793 | 0.6549 | 0.0027 |
| 0.6825 | 0.6554 | 0.0027 |
| 0.6856 | 0.6544 | 0.0027 |
| 0.6888 | 0.6482 | 0.0027 |
| 0.692 | 0.6561 | 0.0027 |
| 0.6952 | 0.6635 | 0.0027 |
| 0.6984 | 0.6506 | 0.0027 |
| 0.7016 | 0.66 | 0.0027 |
| 0.7048 | 0.6505 | 0.0027 |
| 0.7081 | 0.6672 | 0.0027 |
| 0.7114 | 0.6667 | 0.0027 |
| 0.7146 | 0.6572 | 0.0027 |
| 0.7179 | 0.6483 | 0.0027 |
| 0.7213 | 0.6605 | 0.0027 |
| 0.7246 | 0.6504 | 0.0027 |
| 0.7279 | 0.6558 | 0.0027 |
| 0.7313 | 0.656 | 0.0027 |
| 0.7347 | 0.6588 | 0.0027 |
| 0.7381 | 0.6536 | 0.0027 |
| 0.7415 | 0.6522 | 0.0027 |
| 0.7449 | 0.6582 | 0.0027 |
| 0.7483 | 0.6595 | 0.0027 |
| 0.7518 | 0.654 | 0.0027 |
| 0.7552 | 0.653 | 0.0027 |
| 0.7587 | 0.6563 | 0.0027 |
| 0.7622 | 0.6564 | 0.0027 |
| 0.7658 | 0.6589 | 0.0027 |
| 0.7693 | 0.6585 | 0.0027 |
| 0.7728 | 0.6609 | 0.0027 |
| 0.7764 | 0.6603 | 0.0027 |
| 0.78 | 0.6553 | 0.0027 |
| 0.7836 | 0.6581 | 0.0027 |
| 0.7872 | 0.6607 | 0.0027 |
| 0.7908 | 0.6606 | 0.0027 |
| 0.7945 | 0.6529 | 0.0027 |
| 0.7982 | 0.652 | 0.0027 |
| 0.8019 | 0.655 | 0.0027 |
| 0.8056 | 0.6523 | 0.0027 |
| 0.8093 | 0.6618 | 0.0027 |
| 0.813 | 0.6594 | 0.0027 |
| 0.8168 | 0.6571 | 0.0027 |
| 0.8205 | 0.6528 | 0.0027 |
| 0.8243 | 0.6537 | 0.0027 |
| 0.8281 | 0.6611 | 0.0027 |
| 0.8319 | 0.6584 | 0.0027 |
| 0.8358 | 0.6645 | 0.0027 |
| 0.8396 | 0.6605 | 0.0027 |
| 0.8435 | 0.6634 | 0.0027 |
| 0.8474 | 0.6547 | 0.0027 |
| 0.8513 | 0.6669 | 0.0027 |
| 0.8553 | 0.6545 | 0.0027 |
| 0.8592 | 0.6525 | 0.0027 |
| 0.8632 | 0.659 | 0.0027 |
| 0.8672 | 0.6603 | 0.0027 |
| 0.8712 | 0.6513 | 0.0026 |
| 0.8752 | 0.6514 | 0.0027 |
| 0.8792 | 0.6563 | 0.0027 |
| 0.8833 | 0.6584 | 0.0027 |
| 0.8874 | 0.6561 | 0.0027 |
| 0.8915 | 0.6576 | 0.0027 |
| 0.8956 | 0.6569 | 0.0027 |
| 0.8997 | 0.6638 | 0.0027 |
| 0.9039 | 0.6663 | 0.0027 |
| 0.908 | 0.6586 | 0.0027 |
| 0.9122 | 0.6505 | 0.0027 |
| 0.9164 | 0.6554 | 0.0027 |
| 0.9207 | 0.6612 | 0.0027 |
| 0.9249 | 0.6605 | 0.0027 |
| 0.9292 | 0.6602 | 0.0027 |
| 0.9335 | 0.6644 | 0.0027 |
| 0.9378 | 0.6596 | 0.0027 |
| 0.9421 | 0.6519 | 0.0027 |
| 0.9465 | 0.6541 | 0.0027 |
| 0.9508 | 0.6545 | 0.0027 |
| 0.9552 | 0.6556 | 0.0027 |
| 0.9596 | 0.6606 | 0.0027 |
| 0.9641 | 0.6472 | 0.0027 |
| 0.9685 | 0.6533 | 0.0027 |
| 0.973 | 0.6567 | 0.0027 |
| 0.9775 | 0.6554 | 0.0027 |
| 0.982 | 0.6546 | 0.0027 |
| 0.9865 | 0.6559 | 0.0027 |
| 0.9911 | 0.6589 | 0.0027 |
| 0.9957 | 0.6559 | 0.0027 |
| 1.0003 | 0.6603 | 0.0027 |
| 1.0049 | 0.6523 | 0.0027 |
| 1.0095 | 0.6661 | 0.0027 |
| 1.0142 | 0.6563 | 0.0027 |
| 1.0189 | 0.6564 | 0.0027 |
| 1.0236 | 0.6615 | 0.0027 |
| 1.0283 | 0.653 | 0.0027 |
| 1.033 | 0.6644 | 0.0027 |
| 1.0378 | 0.6621 | 0.0027 |
| 1.0426 | 0.6563 | 0.0027 |
| 1.0474 | 0.6647 | 0.0028 |
| 1.0522 | 0.6666 | 0.0027 |
| 1.0571 | 0.6533 | 0.0027 |
| 1.062 | 0.6604 | 0.0027 |
| 1.0669 | 0.66 | 0.0027 |
| 1.0718 | 0.66 | 0.0027 |
| 1.0768 | 0.6531 | 0.0027 |
| 1.0817 | 0.6631 | 0.0028 |
| 1.0867 | 0.6621 | 0.0027 |
| 1.0917 | 0.6591 | 0.0027 |
| 1.0968 | 0.6565 | 0.0027 |
| 1.1018 | 0.6575 | 0.0028 |
| 1.1069 | 0.664 | 0.0028 |
| 1.112 | 0.6581 | 0.0028 |
| 1.1172 | 0.6599 | 0.0028 |
| 1.1223 | 0.6613 | 0.0028 |
| 1.1275 | 0.6562 | 0.0027 |
| 1.1327 | 0.6591 | 0.0028 |
| 1.138 | 0.66 | 0.0028 |
| 1.1432 | 0.6706 | 0.0028 |
| 1.1485 | 0.6598 | 0.0028 |
| 1.1538 | 0.6529 | 0.0027 |
| 1.1591 | 0.6545 | 0.0027 |
| 1.1645 | 0.6616 | 0.0028 |
| 1.1698 | 0.6511 | 0.0027 |
| 1.1752 | 0.6548 | 0.0028 |
| 1.1807 | 0.6513 | 0.0028 |
| 1.1861 | 0.6625 | 0.0028 |
| 1.1916 | 0.6609 | 0.0028 |
| 1.1971 | 0.6551 | 0.0028 |
| 1.2026 | 0.6517 | 0.0028 |
| 1.2082 | 0.6553 | 0.0028 |
| 1.2138 | 0.66 | 0.0028 |
| 1.2193 | 0.6654 | 0.0028 |
| 1.225 | 0.6597 | 0.0028 |
| 1.2306 | 0.6635 | 0.0028 |
| 1.2363 | 0.6527 | 0.0028 |
| 1.242 | 0.6653 | 0.0028 |
| 1.2478 | 0.6542 | 0.0028 |
| 1.2535 | 0.6516 | 0.0028 |
| 1.2593 | 0.6637 | 0.0028 |
| 1.2651 | 0.6688 | 0.0028 |
| 1.271 | 0.6611 | 0.0028 |
| 1.2768 | 0.6607 | 0.0028 |
| 1.2827 | 0.6553 | 0.0028 |
| 1.2887 | 0.6582 | 0.0028 |
| 1.2946 | 0.6531 | 0.0028 |
| 1.3006 | 0.6549 | 0.0028 |
| 1.3066 | 0.6622 | 0.0028 |
| 1.3126 | 0.6437 | 0.0028 |
| 1.3187 | 0.6613 | 0.0028 |
| 1.3248 | 0.6505 | 0.0028 |
| 1.3309 | 0.6658 | 0.0028 |
| 1.337 | 0.6669 | 0.0028 |
| 1.3432 | 0.6523 | 0.0028 |
| 1.3494 | 0.6647 | 0.0029 |
| 1.3556 | 0.658 | 0.0028 |
| 1.3619 | 0.6652 | 0.0028 |
| 1.3682 | 0.6566 | 0.0028 |
| 1.3745 | 0.6506 | 0.0028 |
| 1.3808 | 0.6602 | 0.0028 |
| 1.3872 | 0.6614 | 0.0029 |
| 1.3936 | 0.6579 | 0.0028 |
| 1.4 | 0.6632 | 0.0029 |
| 1.4065 | 0.659 | 0.0029 |
| 1.413 | 0.6584 | 0.0028 |
| 1.4195 | 0.6528 | 0.0028 |
| 1.4261 | 0.6534 | 0.0028 |
| 1.4327 | 0.662 | 0.0029 |
| 1.4393 | 0.6592 | 0.0028 |
| 1.4459 | 0.6541 | 0.0028 |
| 1.4526 | 0.6588 | 0.0029 |
| 1.4593 | 0.6643 | 0.0029 |
| 1.466 | 0.6587 | 0.0029 |
| 1.4728 | 0.6635 | 0.0029 |
| 1.4796 | 0.6572 | 0.0029 |
| 1.4864 | 0.6603 | 0.0029 |
| 1.4933 | 0.6552 | 0.0029 |
| 1.5002 | 0.6576 | 0.0029 |
| 1.5071 | 0.6548 | 0.0029 |
| 1.5141 | 0.661 | 0.0029 |
| 1.5211 | 0.6575 | 0.0029 |
| 1.5281 | 0.6593 | 0.0029 |
| 1.5352 | 0.6512 | 0.0028 |
| 1.5422 | 0.6589 | 0.0029 |
| 1.5494 | 0.6605 | 0.0029 |
| 1.5565 | 0.6568 | 0.0029 |
| 1.5637 | 0.6596 | 0.0029 |
| 1.5709 | 0.6618 | 0.0029 |
| 1.5782 | 0.6591 | 0.0029 |
| 1.5855 | 0.6622 | 0.0029 |
| 1.5928 | 0.652 | 0.0029 |
| 1.6001 | 0.6496 | 0.0029 |
| 1.6075 | 0.6549 | 0.0029 |
| 1.6149 | 0.6568 | 0.0029 |
| 1.6224 | 0.6591 | 0.0029 |
| 1.6299 | 0.6629 | 0.0029 |
| 1.6374 | 0.6548 | 0.0029 |
| 1.645 | 0.6569 | 0.0029 |
| 1.6526 | 0.6649 | 0.0029 |
| 1.6602 | 0.6537 | 0.0029 |
| 1.6679 | 0.663 | 0.0029 |
| 1.6756 | 0.6627 | 0.0029 |
| 1.6833 | 0.6568 | 0.0029 |
| 1.6911 | 0.6599 | 0.0029 |
| 1.6989 | 0.6604 | 0.0029 |
| 1.7067 | 0.6614 | 0.0029 |
| 1.7146 | 0.6635 | 0.0029 |
| 1.7225 | 0.6595 | 0.0029 |
| 1.7305 | 0.6593 | 0.0029 |
| 1.7385 | 0.6621 | 0.0029 |
| 1.7465 | 0.6515 | 0.0029 |
| 1.7546 | 0.6638 | 0.003 |
| 1.7627 | 0.658 | 0.0029 |
| 1.7708 | 0.6544 | 0.0029 |
| 1.779 | 0.6563 | 0.003 |
| 1.7872 | 0.6581 | 0.003 |
| 1.7954 | 0.6481 | 0.0029 |
| 1.8037 | 0.6547 | 0.003 |
| 1.812 | 0.6582 | 0.003 |
| 1.8204 | 0.6592 | 0.003 |
| 1.8288 | 0.6579 | 0.003 |
| 1.8373 | 0.6638 | 0.003 |
| 1.8457 | 0.669 | 0.003 |
| 1.8543 | 0.6539 | 0.003 |
| 1.8628 | 0.6533 | 0.003 |
| 1.8714 | 0.6592 | 0.003 |
| 1.8801 | 0.6595 | 0.003 |
| 1.8887 | 0.6599 | 0.003 |
| 1.8975 | 0.6534 | 0.003 |
| 1.9062 | 0.6617 | 0.003 |
| 1.915 | 0.6535 | 0.003 |
| 1.9239 | 0.656 | 0.003 |
| 1.9328 | 0.6577 | 0.003 |
| 1.9417 | 0.6614 | 0.003 |
| 1.9506 | 0.6584 | 0.003 |
| 1.9596 | 0.6544 | 0.003 |
| 1.9687 | 0.6603 | 0.003 |
| 1.9778 | 0.6632 | 0.003 |
| 1.9869 | 0.6613 | 0.003 |
| 1.9961 | 0.6563 | 0.003 |
| 2.0053 | 0.6522 | 0.003 |
| 2.0146 | 0.6577 | 0.003 |
| 2.0239 | 0.6585 | 0.003 |
| 2.0332 | 0.6569 | 0.003 |
| 2.0426 | 0.6544 | 0.003 |
| 2.052 | 0.6595 | 0.003 |
| 2.0615 | 0.6598 | 0.003 |
| 2.071 | 0.6556 | 0.003 |
| 2.0806 | 0.6558 | 0.003 |
| 2.0902 | 0.6608 | 0.0031 |
| 2.0998 | 0.6563 | 0.003 |
| 2.1095 | 0.6517 | 0.003 |
| 2.1193 | 0.6629 | 0.0031 |
| 2.1291 | 0.6569 | 0.003 |
| 2.1389 | 0.6583 | 0.0031 |
| 2.1488 | 0.659 | 0.003 |
| 2.1587 | 0.6566 | 0.0031 |
| 2.1687 | 0.6599 | 0.003 |
| 2.1787 | 0.6649 | 0.0031 |
| 2.1887 | 0.6534 | 0.0031 |
| 2.1988 | 0.6586 | 0.0031 |
| 2.209 | 0.6661 | 0.0031 |
| 2.2192 | 0.6661 | 0.0031 |
| 2.2294 | 0.6531 | 0.0031 |
| 2.2397 | 0.655 | 0.0031 |
| 2.2501 | 0.6636 | 0.0031 |
| 2.2605 | 0.6678 | 0.0031 |
| 2.2709 | 0.656 | 0.0031 |
| 2.2814 | 0.6631 | 0.0031 |
| 2.2919 | 0.6693 | 0.0031 |
| 2.3025 | 0.6664 | 0.0031 |
| 2.3131 | 0.6614 | 0.0031 |
| 2.3238 | 0.663 | 0.0031 |
| 2.3345 | 0.6572 | 0.0031 |
| 2.3453 | 0.6605 | 0.0031 |
| 2.3561 | 0.6458 | 0.0031 |
| 2.367 | 0.664 | 0.0031 |
| 2.3779 | 0.64 | 0.0031 |
| 2.3889 | 0.6682 | 0.0031 |
| 2.4 | 0.6582 | 0.0031 |
| 2.411 | 0.6662 | 0.0031 |
| 2.4222 | 0.6518 | 0.0031 |
| 2.4333 | 0.6634 | 0.0031 |
| 2.4446 | 0.6652 | 0.0031 |
| 2.4559 | 0.6531 | 0.0031 |
| 2.4672 | 0.6669 | 0.0032 |
| 2.4786 | 0.6588 | 0.0031 |
| 2.4901 | 0.6689 | 0.0032 |
| 2.5015 | 0.6617 | 0.0032 |
| 2.5131 | 0.6566 | 0.0031 |
| 2.5247 | 0.6609 | 0.0032 |
| 2.5364 | 0.6652 | 0.0032 |
| 2.5481 | 0.6638 | 0.0032 |
| 2.5598 | 0.6624 | 0.0032 |
| 2.5717 | 0.6631 | 0.0032 |
| 2.5835 | 0.6529 | 0.0032 |
| 2.5955 | 0.6616 | 0.0032 |
| 2.6074 | 0.6523 | 0.0032 |
| 2.6195 | 0.6548 | 0.0032 |
| 2.6316 | 0.6598 | 0.0032 |
| 2.6437 | 0.6585 | 0.0032 |
| 2.6559 | 0.6571 | 0.0032 |
| 2.6682 | 0.6597 | 0.0032 |
| 2.6805 | 0.6564 | 0.0032 |
| 2.6929 | 0.6553 | 0.0032 |
| 2.7053 | 0.6593 | 0.0032 |
| 2.7178 | 0.6641 | 0.0032 |
| 2.7304 | 0.6571 | 0.0032 |
| 2.743 | 0.6552 | 0.0032 |
| 2.7556 | 0.6545 | 0.0032 |
| 2.7683 | 0.6557 | 0.0032 |
| 2.7811 | 0.6553 | 0.0032 |
| 2.794 | 0.6667 | 0.0032 |
| 2.8069 | 0.6628 | 0.0032 |
| 2.8198 | 0.6604 | 0.0032 |
| 2.8329 | 0.6594 | 0.0032 |
| 2.8459 | 0.6639 | 0.0033 |
| 2.8591 | 0.6646 | 0.0032 |
| 2.8723 | 0.6561 | 0.0033 |
| 2.8855 | 0.657 | 0.0033 |
| 2.8989 | 0.658 | 0.0032 |
| 2.9122 | 0.6521 | 0.0033 |
| 2.9257 | 0.6597 | 0.0033 |
| 2.9392 | 0.658 | 0.0033 |
| 2.9528 | 0.6598 | 0.0032 |
| 2.9664 | 0.6526 | 0.0033 |
| 2.9801 | 0.6566 | 0.0033 |
| 2.9939 | 0.6584 | 0.0033 |
| 3.0077 | 0.6569 | 0.0033 |
| 3.0216 | 0.655 | 0.0033 |
| 3.0355 | 0.6611 | 0.0033 |
| 3.0496 | 0.6607 | 0.0033 |
| 3.0636 | 0.6604 | 0.0033 |
| 3.0778 | 0.6542 | 0.0033 |
| 3.092 | 0.6656 | 0.0033 |
| 3.1063 | 0.6629 | 0.0033 |
| 3.1206 | 0.6572 | 0.0033 |
| 3.135 | 0.6527 | 0.0033 |
| 3.1495 | 0.6506 | 0.0033 |
| 3.164 | 0.6658 | 0.0033 |
| 3.1786 | 0.6697 | 0.0033 |
| 3.1933 | 0.662 | 0.0033 |
| 3.2081 | 0.664 | 0.0033 |
| 3.2229 | 0.6636 | 0.0033 |
| 3.2378 | 0.6529 | 0.0033 |
| 3.2527 | 0.6649 | 0.0033 |
| 3.2677 | 0.6612 | 0.0033 |
| 3.2828 | 0.6667 | 0.0033 |
| 3.298 | 0.6542 | 0.0033 |
| 3.3132 | 0.6556 | 0.0034 |
| 3.3285 | 0.6588 | 0.0033 |
| 3.3439 | 0.6548 | 0.0033 |
| 3.3593 | 0.6578 | 0.0034 |
| 3.3748 | 0.6444 | 0.0033 |
| 3.3904 | 0.6591 | 0.0033 |
| 3.4061 | 0.6643 | 0.0034 |
| 3.4218 | 0.6614 | 0.0034 |
| 3.4376 | 0.659 | 0.0034 |
| 3.4535 | 0.6651 | 0.0034 |
| 3.4694 | 0.6527 | 0.0034 |
| 3.4854 | 0.6604 | 0.0034 |
| 3.5015 | 0.6549 | 0.0034 |
| 3.5177 | 0.6563 | 0.0034 |
| 3.5339 | 0.6596 | 0.0034 |
| 3.5502 | 0.6496 | 0.0034 |
| 3.5666 | 0.6584 | 0.0034 |
| 3.5831 | 0.6643 | 0.0034 |
| 3.5997 | 0.664 | 0.0034 |
| 3.6163 | 0.6556 | 0.0034 |
| 3.633 | 0.6689 | 0.0034 |
| 3.6497 | 0.6622 | 0.0034 |
| 3.6666 | 0.6553 | 0.0034 |
| 3.6835 | 0.6608 | 0.0034 |
| 3.7005 | 0.6612 | 0.0034 |
| 3.7176 | 0.6528 | 0.0034 |
| 3.7348 | 0.652 | 0.0034 |
| 3.752 | 0.6497 | 0.0034 |
| 3.7694 | 0.6537 | 0.0034 |
| 3.7868 | 0.6652 | 0.0034 |
| 3.8043 | 0.6565 | 0.0035 |
| 3.8218 | 0.6657 | 0.0035 |
| 3.8395 | 0.6571 | 0.0034 |
| 3.8572 | 0.6712 | 0.0035 |
| 3.875 | 0.6617 | 0.0035 |
| 3.8929 | 0.6584 | 0.0035 |
| 3.9109 | 0.6568 | 0.0034 |
| 3.9289 | 0.6645 | 0.0035 |
| 3.9471 | 0.6626 | 0.0035 |
| 3.9653 | 0.6439 | 0.0034 |
| 3.9836 | 0.6577 | 0.0035 |
| 4.002 | 0.6551 | 0.0035 |
| 4.0205 | 0.6558 | 0.0035 |
| 4.039 | 0.6582 | 0.0035 |
| 4.0577 | 0.658 | 0.0035 |
| 4.0764 | 0.6734 | 0.0035 |
| 4.0953 | 0.6562 | 0.0035 |
| 4.1142 | 0.6615 | 0.0035 |
| 4.1332 | 0.651 | 0.0035 |
| 4.1523 | 0.6528 | 0.0035 |
| 4.1714 | 0.6565 | 0.0035 |
| 4.1907 | 0.6583 | 0.0035 |
| 4.21 | 0.6631 | 0.0035 |
| 4.2295 | 0.6527 | 0.0035 |
| 4.249 | 0.6584 | 0.0035 |
| 4.2686 | 0.6582 | 0.0035 |
| 4.2883 | 0.6623 | 0.0035 |
| 4.3081 | 0.653 | 0.0035 |
| 4.328 | 0.6492 | 0.0035 |
| 4.348 | 0.6586 | 0.0035 |
| 4.3681 | 0.6581 | 0.0035 |
| 4.3883 | 0.6674 | 0.0036 |
| 4.4085 | 0.6541 | 0.0035 |
| 4.4289 | 0.6612 | 0.0036 |
| 4.4493 | 0.6603 | 0.0036 |
| 4.4699 | 0.6466 | 0.0035 |
| 4.4905 | 0.6616 | 0.0036 |
| 4.5113 | 0.6435 | 0.0035 |
| 4.5321 | 0.6564 | 0.0036 |
| 4.553 | 0.6575 | 0.0036 |
| 4.574 | 0.6465 | 0.0036 |
| 4.5952 | 0.6454 | 0.0036 |
| 4.6164 | 0.6645 | 0.0036 |
| 4.6377 | 0.6476 | 0.0035 |
| 4.6591 | 0.6615 | 0.0036 |
| 4.6806 | 0.6656 | 0.0036 |
| 4.7022 | 0.6586 | 0.0036 |
| 4.724 | 0.6623 | 0.0036 |
| 4.7458 | 0.6659 | 0.0036 |
| 4.7677 | 0.6662 | 0.0036 |
| 4.7897 | 0.6626 | 0.0036 |
| 4.8118 | 0.6535 | 0.0036 |
| 4.834 | 0.6644 | 0.0037 |
| 4.8564 | 0.6634 | 0.0037 |
| 4.8788 | 0.6499 | 0.0036 |
| 4.9013 | 0.6426 | 0.0036 |
| 4.9239 | 0.659 | 0.0036 |
| 4.9467 | 0.6516 | 0.0036 |
| 4.9695 | 0.6575 | 0.0037 |
| 4.9925 | 0.6432 | 0.0036 |
| 5.0155 | 0.6604 | 0.0037 |
| 5.0387 | 0.6563 | 0.0036 |
| 5.062 | 0.6545 | 0.0037 |
| 5.0853 | 0.6523 | 0.0036 |
| 5.1088 | 0.6561 | 0.0037 |
| 5.1324 | 0.643 | 0.0036 |
| 5.1561 | 0.6624 | 0.0037 |
| 5.1799 | 0.6587 | 0.0037 |
| 5.2038 | 0.6482 | 0.0037 |
| 5.2279 | 0.649 | 0.0037 |
| 5.252 | 0.6511 | 0.0037 |
| 5.2763 | 0.6458 | 0.0037 |
| 5.3006 | 0.6465 | 0.0037 |
| 5.3251 | 0.6555 | 0.0037 |
| 5.3497 | 0.6415 | 0.0037 |
| 5.3744 | 0.6644 | 0.0037 |
| 5.3992 | 0.6499 | 0.0037 |
| 5.4242 | 0.6545 | 0.0037 |
| 5.4492 | 0.6511 | 0.0037 |
| 5.4744 | 0.6581 | 0.0037 |
| 5.4996 | 0.6455 | 0.0037 |
| 5.525 | 0.6418 | 0.0037 |
| 5.5506 | 0.6473 | 0.0037 |
| 5.5762 | 0.6561 | 0.0037 |
| 5.6019 | 0.6537 | 0.0037 |
| 5.6278 | 0.6515 | 0.0037 |
| 5.6538 | 0.6506 | 0.0038 |
| 5.6799 | 0.6486 | 0.0037 |
| 5.7061 | 0.6592 | 0.0038 |
| 5.7325 | 0.645 | 0.0037 |
| 5.7589 | 0.6559 | 0.0038 |
| 5.7855 | 0.6409 | 0.0037 |
| 5.8123 | 0.6326 | 0.0037 |
| 5.8391 | 0.6276 | 0.0037 |
| 5.8661 | 0.6346 | 0.0038 |
| 5.8932 | 0.6331 | 0.0037 |
| 5.9204 | 0.6307 | 0.0037 |
| 5.9477 | 0.6114 | 0.0037 |
| 5.9752 | 0.6083 | 0.0037 |
| 6.0028 | 0.5877 | 0.0036 |
| 6.0305 | 0.5593 | 0.0036 |
| 6.0583 | 0.5297 | 0.0035 |
| 6.0863 | 0.4721 | 0.0033 |
| 6.1144 | 0.3972 | 0.0031 |
| 6.1427 | 0.3115 | 0.0029 |
| 6.171 | 0.2377 | 0.0026 |
| 6.1995 | 0.1821 | 0.0024 |
| 6.2282 | 0.1808 | 0.0024 |
| 6.2569 | 0.2081 | 0.0025 |
| 6.2858 | 0.275 | 0.0027 |
| 6.3148 | 0.3408 | 0.003 |
| 6.344 | 0.43 | 0.0032 |
| 6.3733 | 0.4983 | 0.0035 |
| 6.4027 | 0.5661 | 0.0037 |
| 6.4323 | 0.591 | 0.0037 |
| 6.462 | 0.6137 | 0.0038 |
| 6.4918 | 0.6084 | 0.0038 |
| 6.5218 | 0.6287 | 0.0038 |
| 6.5519 | 0.6326 | 0.0038 |
| 6.5822 | 0.6352 | 0.0038 |
| 6.6126 | 0.6374 | 0.0038 |
| 6.6431 | 0.6441 | 0.0039 |
| 6.6738 | 0.642 | 0.0038 |
| 6.7046 | 0.6512 | 0.0038 |
| 6.7356 | 0.6586 | 0.0039 |
| 6.7667 | 0.6458 | 0.0039 |
| 6.798 | 0.6451 | 0.0039 |
| 6.8293 | 0.649 | 0.0039 |
| 6.8609 | 0.6565 | 0.0039 |
| 6.8926 | 0.6641 | 0.0039 |
| 6.9244 | 0.6569 | 0.0039 |
| 6.9564 | 0.6571 | 0.004 |
| 6.9885 | 0.6515 | 0.0039 |
| 7.0208 | 0.6629 | 0.004 |
| 7.0532 | 0.6377 | 0.0039 |
| 7.0858 | 0.6656 | 0.004 |
| 7.1185 | 0.6487 | 0.004 |
| 7.1514 | 0.6588 | 0.004 |
| 7.1844 | 0.6435 | 0.0039 |
| 7.2176 | 0.66 | 0.004 |
| 7.2509 | 0.6484 | 0.004 |
| 7.2844 | 0.6615 | 0.004 |
| 7.318 | 0.6426 | 0.004 |
| 7.3518 | 0.6491 | 0.004 |
| 7.3858 | 0.6568 | 0.004 |
| 7.4199 | 0.6589 | 0.004 |
| 7.4542 | 0.6497 | 0.004 |
| 7.4886 | 0.662 | 0.0041 |
| 7.5232 | 0.6552 | 0.004 |
| 7.5579 | 0.6636 | 0.004 |
| 7.5928 | 0.661 | 0.004 |
| 7.6279 | 0.6572 | 0.004 |
| 7.6631 | 0.6495 | 0.004 |
| 7.6985 | 0.6465 | 0.004 |
| 7.734 | 0.6561 | 0.004 |
| 7.7698 | 0.6388 | 0.004 |
| 7.8057 | 0.6745 | 0.0041 |
| 7.8417 | 0.6533 | 0.004 |
| 7.8779 | 0.6719 | 0.0041 |
| 7.9143 | 0.6609 | 0.0041 |
| 7.9508 | 0.653 | 0.004 |
| 7.9876 | 0.6566 | 0.004 |
| 8.0244 | 0.6682 | 0.0041 |
| 8.0615 | 0.6599 | 0.0041 |
| 8.0987 | 0.6659 | 0.0041 |
| 8.1361 | 0.6668 | 0.0041 |
| 8.1737 | 0.6596 | 0.0041 |
| 8.2115 | 0.6625 | 0.0041 |
| 8.2494 | 0.6595 | 0.0041 |
| 8.2875 | 0.6546 | 0.0041 |
| 8.3258 | 0.6608 | 0.0041 |
| 8.3642 | 0.6646 | 0.0041 |
| 8.4028 | 0.6674 | 0.0042 |
| 8.4416 | 0.6524 | 0.0041 |
| 8.4806 | 0.6544 | 0.0041 |
| 8.5198 | 0.6651 | 0.0042 |
| 8.5592 | 0.6603 | 0.0041 |
| 8.5987 | 0.657 | 0.0041 |
| 8.6384 | 0.6624 | 0.0042 |
| 8.6783 | 0.6648 | 0.0042 |
| 8.7184 | 0.6508 | 0.0041 |
| 8.7586 | 0.6604 | 0.0042 |
| 8.7991 | 0.6474 | 0.0041 |
| 8.8397 | 0.6494 | 0.0041 |
| 8.8805 | 0.6458 | 0.0042 |
| 8.9216 | 0.6557 | 0.0042 |
| 8.9628 | 0.6689 | 0.0042 |
| 9.0042 | 0.6604 | 0.0042 |
| 9.0457 | 0.6577 | 0.0042 |
| 9.0875 | 0.6515 | 0.0042 |
| 9.1295 | 0.6659 | 0.0042 |
| 9.1717 | 0.6526 | 0.0042 |
| 9.214 | 0.6613 | 0.0042 |
| 9.2566 | 0.6581 | 0.0043 |
| 9.2993 | 0.6569 | 0.0042 |
| 9.3423 | 0.64 | 0.0042 |
| 9.3854 | 0.6752 | 0.0043 |
| 9.4288 | 0.6689 | 0.0043 |
| 9.4723 | 0.6593 | 0.0043 |
| 9.5161 | 0.66 | 0.0043 |
| 9.56 | 0.6606 | 0.0042 |
| 9.6042 | 0.6575 | 0.0043 |
| 9.6485 | 0.6472 | 0.0042 |
| 9.6931 | 0.6639 | 0.0043 |
| 9.7378 | 0.6608 | 0.0043 |
| 9.7828 | 0.6438 | 0.0043 |
| 9.828 | 0.6514 | 0.0043 |
| 9.8734 | 0.6558 | 0.0043 |
| 9.919 | 0.6806 | 0.0044 |
| 9.9648 | 0.6623 | 0.0043 |
| 10.0108 | 0.6594 | 0.0043 |
| 10.0571 | 0.647 | 0.0042 |
| 10.1035 | 0.6564 | 0.0043 |
| 10.1502 | 0.6508 | 0.0043 |
| 10.1971 | 0.6584 | 0.0043 |
| 10.2442 | 0.6776 | 0.0044 |
| 10.2915 | 0.66 | 0.0043 |
| 10.339 | 0.6662 | 0.0044 |
| 10.3868 | 0.6606 | 0.0043 |
| 10.4347 | 0.659 | 0.0044 |
| 10.4829 | 0.6425 | 0.0043 |
| 10.5313 | 0.6608 | 0.0044 |
| 10.58 | 0.6767 | 0.0044 |
| 10.6288 | 0.6772 | 0.0044 |
| 10.6779 | 0.657 | 0.0043 |
| 10.7273 | 0.6654 | 0.0044 |
| 10.7768 | 0.6608 | 0.0044 |
| 10.8266 | 0.6751 | 0.0044 |
| 10.8766 | 0.6674 | 0.0044 |
| 10.9268 | 0.6459 | 0.0044 |
| 10.9773 | 0.6575 | 0.0044 |
| 11.028 | 0.6513 | 0.0044 |
| 11.0789 | 0.6502 | 0.0044 |
| 11.1301 | 0.6491 | 0.0044 |
| 11.1815 | 0.6641 | 0.0044 |
| 11.2331 | 0.6525 | 0.0044 |
| 11.285 | 0.6648 | 0.0044 |
| 11.3372 | 0.6479 | 0.0044 |
| 11.3895 | 0.6373 | 0.0044 |
| 11.4421 | 0.661 | 0.0045 |
| 11.495 | 0.6625 | 0.0045 |
| 11.5481 | 0.665 | 0.0045 |
| 11.6014 | 0.653 | 0.0045 |
| 11.655 | 0.6742 | 0.0045 |
| 11.7088 | 0.6569 | 0.0045 |
| 11.7629 | 0.6474 | 0.0045 |
| 11.8172 | 0.655 | 0.0045 |
| 11.8718 | 0.6566 | 0.0045 |
| 11.9266 | 0.6661 | 0.0045 |
| 11.9817 | 0.6394 | 0.0045 |
| 12.0371 | 0.6491 | 0.0045 |
| 12.0927 | 0.6606 | 0.0045 |
| 12.1485 | 0.6451 | 0.0045 |
| 12.2046 | 0.6551 | 0.0045 |
| 12.261 | 0.6563 | 0.0046 |
| 12.3176 | 0.6519 | 0.0045 |
| 12.3745 | 0.6865 | 0.0046 |
| 12.4317 | 0.671 | 0.0046 |
| 12.4891 | 0.6578 | 0.0046 |
| 12.5468 | 0.6678 | 0.0046 |
| 12.6048 | 0.6743 | 0.0046 |
| 12.663 | 0.6538 | 0.0046 |
| 12.7215 | 0.6433 | 0.0045 |
| 12.7802 | 0.6583 | 0.0046 |
| 12.8393 | 0.6503 | 0.0046 |
| 12.8986 | 0.6685 | 0.0046 |
| 12.9581 | 0.6571 | 0.0046 |
| 13.018 | 0.6526 | 0.0046 |
| 13.0781 | 0.6602 | 0.0046 |
| 13.1385 | 0.6664 | 0.0047 |
| 13.1992 | 0.6564 | 0.0046 |
| 13.2602 | 0.659 | 0.0046 |
| 13.3214 | 0.6638 | 0.0047 |
| 13.383 | 0.6713 | 0.0047 |
| 13.4448 | 0.6645 | 0.0047 |
| 13.5069 | 0.6518 | 0.0046 |
| 13.5693 | 0.6317 | 0.0046 |
| 13.6319 | 0.6639 | 0.0047 |
| 13.6949 | 0.6574 | 0.0046 |
| 13.7582 | 0.6549 | 0.0047 |
| 13.8217 | 0.6601 | 0.0046 |
| 13.8856 | 0.6425 | 0.0046 |
| 13.9497 | 0.6656 | 0.0047 |
| 14.0141 | 0.6542 | 0.0047 |
| 14.0789 | 0.6508 | 0.0047 |
| 14.1439 | 0.6453 | 0.0046 |
| 14.2092 | 0.6583 | 0.0047 |
| 14.2749 | 0.6474 | 0.0046 |
| 14.3408 | 0.6593 | 0.0048 |
| 14.407 | 0.6648 | 0.0047 |
| 14.4736 | 0.6525 | 0.0047 |
| 14.5405 | 0.6434 | 0.0047 |
| 14.6076 | 0.6725 | 0.0048 |
| 14.6751 | 0.6359 | 0.0047 |
| 14.7429 | 0.6247 | 0.0047 |
| 14.811 | 0.6396 | 0.0047 |
| 14.8794 | 0.6317 | 0.0047 |
| 14.9481 | 0.628 | 0.0047 |
| 15.0172 | 0.611 | 0.0047 |
| 15.0865 | 0.5803 | 0.0046 |
| 15.1562 | 0.5342 | 0.0044 |
| 15.2262 | 0.4359 | 0.0041 |
| 15.2966 | 0.3272 | 0.0037 |
| 15.3672 | 0.2329 | 0.0034 |
| 15.4382 | 0.2487 | 0.0034 |
| 15.5095 | 0.3354 | 0.0038 |
| 15.5812 | 0.4569 | 0.0042 |
| 15.6531 | 0.5501 | 0.0045 |
| 15.7255 | 0.6141 | 0.0047 |
| 15.7981 | 0.6333 | 0.0048 |
| 15.8711 | 0.6591 | 0.0048 |
| 15.9444 | 0.6467 | 0.0048 |
| 16.018 | 0.6635 | 0.0049 |
| 16.092 | 0.6458 | 0.0048 |
| 16.1664 | 0.6571 | 0.0049 |
| 16.241 | 0.6263 | 0.0047 |
| 16.3161 | 0.6512 | 0.0048 |
| 16.3914 | 0.6598 | 0.0049 |
| 16.4672 | 0.6535 | 0.0049 |
| 16.5432 | 0.6691 | 0.0049 |
| 16.6196 | 0.6516 | 0.0049 |
| 16.6964 | 0.6655 | 0.0049 |
| 16.7735 | 0.6751 | 0.005 |
| 16.851 | 0.6602 | 0.0049 |
| 16.9289 | 0.6439 | 0.0049 |
| 17.0071 | 0.6483 | 0.0049 |
| 17.0856 | 0.6497 | 0.0049 |
| 17.1646 | 0.6608 | 0.005 |
| 17.2438 | 0.6526 | 0.005 |
| 17.3235 | 0.6677 | 0.005 |
| 17.4035 | 0.6618 | 0.0049 |
| 17.4839 | 0.6585 | 0.0049 |
| 17.5647 | 0.6568 | 0.0049 |
| 17.6458 | 0.6499 | 0.0049 |
| 17.7273 | 0.6585 | 0.005 |
| 17.8092 | 0.6409 | 0.0049 |
| 17.8915 | 0.6642 | 0.005 |
| 17.9742 | 0.6612 | 0.005 |
| 18.0572 | 0.6513 | 0.005 |
| 18.1406 | 0.6448 | 0.005 |
| 18.2244 | 0.6585 | 0.005 |
| 18.3086 | 0.663 | 0.0052 |
| 18.3932 | 0.6539 | 0.0052 |
| 18.4781 | 0.6507 | 0.005 |
| 18.5635 | 0.6399 | 0.0051 |
| 18.6493 | 0.6686 | 0.0051 |
| 18.7354 | 0.6616 | 0.005 |
| 18.822 | 0.6571 | 0.0051 |
| 18.9089 | 0.6332 | 0.005 |
| 18.9963 | 0.6497 | 0.005 |
| 19.084 | 0.6821 | 0.0051 |
| 19.1722 | 0.649 | 0.0051 |
| 19.2608 | 0.6622 | 0.0051 |
| 19.3497 | 0.6578 | 0.0051 |
| 19.4391 | 0.6564 | 0.0051 |
| 19.5289 | 0.6449 | 0.0051 |
| 19.6191 | 0.6481 | 0.0051 |
| 19.7098 | 0.6635 | 0.0051 |
| 19.8008 | 0.6516 | 0.0051 |
| 19.8923 | 0.6487 | 0.0052 |
| 19.9842 | 0.6451 | 0.0051 |
| 20.0765 | 0.6445 | 0.005 |
| 20.1693 | 0.6559 | 0.0051 |
| 20.2624 | 0.6431 | 0.005 |
| 20.3561 | 0.6577 | 0.0052 |
| 20.4501 | 0.65 | 0.0051 |
| 20.5446 | 0.6453 | 0.0051 |
| 20.6395 | 0.6377 | 0.0051 |
| 20.7348 | 0.6173 | 0.0051 |
| 20.8306 | 0.6085 | 0.0051 |
| 20.9269 | 0.6035 | 0.005 |
| 21.0235 | 0.5583 | 0.0049 |
| 21.1207 | 0.4719 | 0.0046 |
| 21.2182 | 0.324 | 0.0041 |
| 21.3163 | 0.1515 | 0.0033 |
| 21.4147 | 0.1064 | 0.0031 |
| 21.5137 | 0.1596 | 0.0033 |
| 21.6131 | 0.2983 | 0.0039 |
| 21.7129 | 0.4687 | 0.0046 |
| 21.8132 | 0.5777 | 0.005 |
| 21.914 | 0.613 | 0.0051 |
| 22.0152 | 0.6074 | 0.0051 |
| 22.117 | 0.6307 | 0.0051 |
| 22.2191 | 0.6418 | 0.0053 |
| 22.3218 | 0.6304 | 0.0052 |
| 22.4249 | 0.6532 | 0.0053 |
| 22.5285 | 0.6349 | 0.0052 |
| 22.6326 | 0.6508 | 0.0053 |
| 22.7372 | 0.6531 | 0.0052 |
| 22.8422 | 0.6548 | 0.0054 |
| 22.9477 | 0.64 | 0.0052 |
| 23.0538 | 0.6559 | 0.0053 |
| 23.1603 | 0.6382 | 0.0053 |
| 23.2673 | 0.6544 | 0.0053 |
| 23.3748 | 0.6462 | 0.0053 |
| 23.4828 | 0.644 | 0.0053 |
| 23.5912 | 0.6576 | 0.0054 |
| 23.7002 | 0.6618 | 0.0054 |
| 23.8097 | 0.6547 | 0.0054 |
| 23.9197 | 0.6705 | 0.0054 |
| 24.0303 | 0.6544 | 0.0054 |
| 24.1413 | 0.6507 | 0.0053 |
| 24.2528 | 0.6476 | 0.0054 |
| 24.3649 | 0.6435 | 0.0054 |
| 24.4774 | 0.6521 | 0.0054 |
| 24.5905 | 0.6657 | 0.0055 |
| 24.7041 | 0.6581 | 0.0054 |
| 24.8183 | 0.6561 | 0.0055 |
| 24.9329 | 0.6532 | 0.0055 |
| 25.0481 | 0.653 | 0.0054 |
| 25.1639 | 0.6677 | 0.0055 |
| 25.2801 | 0.6511 | 0.0055 |
| 25.3969 | 0.6645 | 0.0055 |
| 25.5143 | 0.6527 | 0.0055 |
| 25.6321 | 0.6703 | 0.0055 |
| 25.7506 | 0.6862 | 0.0056 |
| 25.8696 | 0.6702 | 0.0055 |
| 25.9891 | 0.6616 | 0.0056 |
| 26.1092 | 0.6771 | 0.0056 |
| 26.2298 | 0.6571 | 0.0055 |
| 26.351 | 0.6674 | 0.0056 |
| 26.4727 | 0.6597 | 0.0056 |
| 26.595 | 0.6675 | 0.0055 |
| 26.7179 | 0.6472 | 0.0055 |
| 26.8414 | 0.6438 | 0.0055 |
| 26.9654 | 0.6656 | 0.0057 |
| 27.09 | 0.6891 | 0.0058 |
| 27.2151 | 0.6631 | 0.0059 |
| 27.3409 | 0.6631 | 0.0062 |
| 27.4672 | 0.6241 | 0.0065 |
| 27.5941 | 0.6757 | 0.0074 |
| 27.7216 | 0.6647 | 0.0071 |
| 27.8497 | 0.6461 | 0.0063 |
| 27.9784 | 0.6375 | 0.0057 |
| 28.1077 | 0.6706 | 0.0058 |
| 28.2375 | 0.6738 | 0.0057 |
| 28.368 | 0.6729 | 0.0057 |
| 28.4991 | 0.6422 | 0.0056 |
| 28.6308 | 0.6495 | 0.0058 |
| 28.763 | 0.6402 | 0.0056 |
| 28.8959 | 0.6469 | 0.0056 |
| 29.0295 | 0.666 | 0.0058 |
| 29.1636 | 0.6372 | 0.0056 |
| 29.2983 | 0.6472 | 0.0056 |
| 29.4337 | 0.5957 | 0.0055 |
| 29.5697 | 0.5141 | 0.0053 |
| 29.7063 | 0.4992 | 0.005 |
| 29.8436 | 0.5713 | 0.0054 |
| 29.9815 | 0.6294 | 0.0057 |
| 30.12 | 0.6503 | 0.0057 |
| 30.2592 | 0.6647 | 0.0056 |
| 30.399 | 0.6683 | 0.0058 |
| 30.5395 | 0.6657 | 0.0057 |
| 30.6806 | 0.6542 | 0.0058 |
| 30.8224 | 0.6616 | 0.0057 |
| 30.9648 | 0.6583 | 0.0057 |
| 31.1079 | 0.6465 | 0.0058 |
| 31.2516 | 0.6734 | 0.0059 |
| 31.396 | 0.6607 | 0.0058 |
| 31.5411 | 0.6558 | 0.0058 |
| 31.6868 | 0.6524 | 0.0057 |
| 31.8333 | 0.6562 | 0.0059 |
| 31.9803 | 0.6659 | 0.0058 |
| 32.1281 | 0.6583 | 0.0059 |
| 32.2766 | 0.6616 | 0.0058 |
| 32.4257 | 0.6486 | 0.0058 |
| 32.5756 | 0.67 | 0.0059 |
| 32.7261 | 0.6547 | 0.0059 |
| 32.8773 | 0.6611 | 0.0058 |
| 33.0292 | 0.6644 | 0.0059 |
| 33.1819 | 0.6561 | 0.0059 |
| 33.3352 | 0.6616 | 0.006 |
| 33.4892 | 0.6404 | 0.0058 |
| 33.644 | 0.6559 | 0.0059 |
| 33.7994 | 0.6437 | 0.0058 |
| 33.9556 | 0.673 | 0.0059 |
| 34.1125 | 0.6665 | 0.006 |
| 34.2702 | 0.6646 | 0.0059 |
| 34.4285 | 0.6643 | 0.0058 |
| 34.5876 | 0.6516 | 0.006 |
| 34.7474 | 0.6642 | 0.006 |
| 34.908 | 0.667 | 0.006 |
| 35.0693 | 0.6628 | 0.006 |
| 35.2314 | 0.667 | 0.006 |
| 35.3942 | 0.6737 | 0.0061 |
| 35.5577 | 0.6673 | 0.0061 |
| 35.7221 | 0.6599 | 0.006 |
| 35.8871 | 0.6581 | 0.0061 |
| 36.053 | 0.6636 | 0.0061 |
| 36.2196 | 0.671 | 0.0061 |
| 36.3869 | 0.6735 | 0.0061 |
| 36.5551 | 0.6569 | 0.0061 |
| 36.724 | 0.6559 | 0.0061 |
| 36.8937 | 0.6683 | 0.0061 |
| 37.0642 | 0.6324 | 0.0059 |
| 37.2355 | 0.6537 | 0.006 |
| 37.4076 | 0.6568 | 0.0062 |
| 37.5804 | 0.6652 | 0.0061 |
| 37.7541 | 0.694 | 0.0061 |
| 37.9286 | 0.6618 | 0.0061 |
| 38.1039 | 0.6538 | 0.0061 |
| 38.2799 | 0.6596 | 0.0061 |
| 38.4568 | 0.6662 | 0.0063 |
| 38.6346 | 0.6723 | 0.0061 |
| 38.8131 | 0.6704 | 0.0061 |
| 38.9925 | 0.6591 | 0.0062 |
| 39.1727 | 0.6525 | 0.006 |
| 39.3537 | 0.6491 | 0.0061 |
| 39.5356 | 0.6633 | 0.0062 |
| 39.7183 | 0.6612 | 0.0063 |
| 39.9018 | 0.6554 | 0.0062 |
| 40.0862 | 0.6569 | 0.0061 |
