# Supplementary material for: Production of zirconium-88 via proton irradiation of metallic yttrium and preparation of target for neutron transmission measurements at DICER
Source: Sci Rep. 2023 Jan 31;13:1736. doi: 10.1038/s41598-023-27993-7 (PMC9889377; doi:10.1038/s41598-023-27993-7)
Supplement: Supplementary file 3 — Supplementary Information 2. [file 41598_2023_27993_MOESM3_ESM.docx]

Suppletory information file includes figures of unsealed (Fig. S1) and sealed (Fig. S2) ^88^Zr sample can using Pb sphere and lever press inside a hot cell and measured DICER neutron transmission data through ^nat^Zr target sample (1.4 µg) in 8 µL of CCl_4_ with 0.3 ·mol·L^−1^ TTA and Pb windows (Table S1) and in 8 µL of 2 ·mol·L^−1^ DCl in D_2_O (with 99.95% D atoms) and Pb windows (Table S2)

Supplementary video shows dispensing of 8 µL sample into tungsten sample can with 1.2 mm diameter inside a hot cell using automated station and Hamilton syringe.
